# Retention in RCTs of physical rehabilitation for adults with frailty: a systematic review and meta-analysis

**DOI:** 10.1186/s13063-022-06172-5

**Published:** 2022-03-28

**Authors:** Heather K. O’Grady, Christopher Farley, Alyson Takaoka, Elisa Mayens, Jackie Bosch, Lyn Turkstra, Michelle E. Kho

**Affiliations:** 1 School of Rehabilitation Sciences, Faculty of Health Sciences, McMaster University, Institute of Applied Health Sciences, 1400 Main St. W., Hamilton, ON L8S 1C7 Canada; 2 Department of Health Research Methods, Evidence, and Impact, Faculty of Health Sciences, McMaster University, Hamilton, ON Canada; 3 Physiotherapy Department, St. Joseph’s Healthcare, Hamilton, ON Canada

**Keywords:** Frailty, Physical rehabilitation, Physiotherapy, Retention, Randomized controlled trials

## Abstract

**Background:**

Physical rehabilitation (PR) interventions can improve physical function for adults with frailty; however, participant retention rates in randomized controlled trials (RCTs) are unknown.

*Objective* is to summarize participant retention rates in RCTs of PR for adults with frailty.

*Design* is a systematic review and meta-analysis (DOI:10.17605/OSF.IO/G6XR2).

*Participants* are adults ≥ 18 years with frailty.

*Setting* consists of inpatient, outpatient and community-based interventions.

*Intervention* includes any PR intervention.

**Methods:**

We searched 7 electronic databases from inception to April 15, 2020 for published RCTs. Our primary outcome was participant retention rate to primary outcome measurement. Secondary outcomes included retention by study group, participant retention to intervention completion, reported reasons for attrition and reported strategies for maximizing retention. We completed screening, data extraction and risk of bias (ROB) assessments independently and in duplicate. We conducted a meta-analysis, calculating retention rates and 95% confidence intervals (CIs) using fixed or random-effects models, as appropriate.

**Results:**

We included 21 RCTs, enrolling 1685 adults with frailty (median age 82.5 years (79.0, 82.2), 59.8% female (57.5, 69.8)). Twenty RCTs reported retention data, of which 90.0% (*n* = 18) had high ROB. The pooled participant retention rate to primary outcome measurement was 85.0% [95%CI (80.0, 90.0), *I*^2^ = 83.9%, *p* < 0.05]. There were no differences by group for retention to the primary outcome [intervention 87.0% (83.0, 91.0), *p* < 0.05, comparator 85.0% (79.0, 90.0), *p* < 0.05] or in retention to intervention completion [83.0% (95.0% CI (78.0–87.0), *p* < 0.05]. Of the 18 studies reporting 24 reasons for attrition, 51.3% were categorized as potentially modifiable by the research team (e.g. low motivation). Only 20.0% (*n* = 4) of studies reported strategies for maximizing retention.

**Conclusions:**

In this review of 21 RCTs of PR, we identified acceptable rates of retention for adults with frailty. High retention in PR interventions appears to be feasible in this population; however, our results are limited by a high ROB and heterogeneity.

**Supplementary Information:**

The online version contains supplementary material available at 10.1186/s13063-022-06172-5.

## Introduction

Frailty is a clinical syndrome characterized by decline in several physiological systems and increased vulnerability to external stressors [1]. Frailty is associated with decreased physical function, both of which are associated with an increased risk of negative outcomes such as falls, hospitalization, disability or death [2, 3]. Negative outcomes associated with frailty have important implications, including decreased quality of life among persons with frailty and their caregivers, increased healthcare spending and increased use of healthcare resources [4–6]. It is becoming increasingly important to identify effective interventions to improve physical function and decrease negative outcomes for the growing number of adults with frailty.

Physical rehabilitation (PR) can improve physical function for adults with frailty [7]. A systematic review of 8 randomized controlled trials (RCTs) and 1068 adults with frailty documented that exercise interventions increased gait speed [+ 0.07, 95% confidence interval (CI) (0.02, 0.11) m/s], improved Berg Balance scores [+ 1.7 (0.60, 2.8) points] and improved activities of daily living across 3 measures [weighted mean difference 5.3 (1.0, 9.6)] compared to control groups [8]. However, of the 8 studies, 3 RCTs enrolling 240 patients reported overall study retention rates of 73–77%, representing high attrition bias according to the Physiotherapy Evidence Database (PEDro) scale for rating quality of RCTs [9].

### Research gap

While the results of PR trials could improve functional outcomes for adults with frailty, retention is a concern. Participant retention has important implications for clinical trial design, conduct, data analysis and results [10].

Our primary objective was to estimate retention rates (from randomization to primary outcome measurement) of adults (≥ 18 years) with frailty enrolled in RCTs of PR. Our secondary objectives were to (1) compare retention rates by group (intervention vs. control) (2); estimate retention rates from randomization to intervention completion (3); summarize reported reasons for attrition (loss of participants from an RCT [11]) and (4) summarize reported strategies for maximizing retention.

## Methods

We prospectively registered this review in Open Science Framework (DOI:10.17605/OSF.IO/G6XR2) [12] and followed the Preferred Reporting Items for Systematic Review and Meta-Analysis (PRISMA) Statement [13] (PRISMA checklist, Supplementary Table S1).

### Inclusion and exclusion criteria

Full details of inclusion and exclusion criteria are published in our protocol (Supplementary Table S2) [12]. Briefly, we included RCTs enrolling adults (≥ 18 years) with frailty (identified in each RCT as an inclusion criterion and assessed using a standardized tool or measure). We included RCTs enrolling adults with pre-frailty if > 50% had frailty or results were presented independently for participants with frailty. We included studies of any PR intervention delivered with the intent to enhance or restore physical function, delivered by a healthcare professional (e.g. exercise programs or modalities) [14], with any comparator group. We excluded conference abstracts, non-English publications, studies and study arms with a multicomponent intervention with another intervention other than PR (e.g. nutrition and PR), and studies of pre-surgical exercise (increased patient motivation in this population [15] may contribute to decreased attrition) [16].

### Search strategy

We identified 7 relevant electronic databases in consultation with a Health Research Librarian: OVID Medline Epub Ahead of Print, In-Process & Other Non-Indexed Citations, Ovid MEDLINE(R) Daily and Ovid MEDLINE(R), Ovid EMBASE, The Cumulative Index to Nursing and Allied Health Literature (CINAHL), Ageline, Web of Science, Allied and Complementary Medicine Database (AMED) and the Cochrane Library. We developed and pilot-tested unique search strategies for each database (Supplementary Table S3). To leverage known systematic reviews of RCTs evaluating PR efficacy, we used a two-stage search approach to identify relevant RCTs.

#### Stage 1: Identification of systematic reviews with potentially relevant RCTs

We searched for systematic reviews of RCTs as a source for potentially relevant RCTs. We searched for the concepts “systematic review”, “adults with frailty” and “physical rehabilitation”. For the concept “systematic review”, we used validated search terms developed for Ovid MEDLINE, EMBASE and CINAHL [17]. For Ageline, Web of Science and AMED, we developed search terms using published search strategies for overviews of systematic reviews in these databases. Search terms were not necessary for systematic reviews in Cochrane. We developed unique terms for “adults with frailty” and “physical rehabilitation”, using similar terms across databases [12].

We searched all databases from inception to December 31, 2019. A study was deemed a “systematic review” if the authors stated use of the systematic review methodology, and reported key components of the search and selection process (e.g. literature sources, search strategy, inclusion/exclusion criteria, screening methods) [18].

#### Stage 2: Identification of primary RCTs

- From systematic reviews, we reviewed RCTs for inclusion.
- We then sought newer primary RCTs. We identified the most recent search end-date from stage 2a (July 1, 2019) and searched all databases until April 15, 2020. We searched for the concepts “randomized controlled trial”, “adults with frailty” and “physical rehabilitation”. To identify RCTs, we used validated search terms from Cochrane and The University of Alberta for each database [18, 19].
- We searched ClinicalTrials.gov and The International Standard Randomised Controlled Trial Number Registry for results of unpublished studies.

### Study selection, data extraction and quality assessment

We used Covidence (2020, Melbourne, Australia: Veritas Health Innovation) for study selection, data extraction and quality assessment. Two reviewers screened citations by title/abstract (HKO, CF) and full-text independently and in duplicate (HKO, CF/EM). Disagreements were solved by consensus and we consulted a third reviewer (MEK) if necessary. We assessed reviewer agreement at each stage using proportionate agreement and Cohen’s kappa (*κ*) [20].

Data extraction and quality assessment were completed independently and in duplicate by two reviewers (HKO, CF/AT). We assessed quality using the Cochrane Risk of Bias (ROB) 2.0 tool for ROB in RCTs [21]. While ROB 2.0 is outcome specific, each source of bias has the potential to affect our estimates of retention. We assessed ROB as high, some concern, or low, according to the Cochrane Handbook [18]. We developed and pilot-tested a data extraction and quality assessment sheet. We completed calibration by each extracting data and assessing quality for two studies (10%) and reviewing as a team [22]. Our data points are summarized in Supplementary Table S4. We extracted retention data for the pre-specified primary outcome and timepoint of each RCT included. If a study’s primary outcome or timepoint were not specified, we used the outcome or timepoint closest to intervention completion for our retention rate calculation [23]. For our primary objective, we defined retention as the proportion of participants who provided primary outcome data out of all randomized participants, irrespective of intervention adherence [11, 23, 24]. For our secondary objective, we calculated the proportion of participants who completed the intervention, out of all randomized.

For randomized cross-over trials, we extracted data from intervention and comparator groups for the first phase of the study. For cluster RCTs, since the unit of randomization was a group of participants rather than an individual participant, our unit of analysis was the randomized group [18]. If cluster RCTs reported individual participants as the unit of analysis, we calculated effective sample sizes for the number of participants enrolled and retained [18, 25].

### Statistical analysis

Data were analysed using Stata (v. 15.0, College Station, Texas: StataCorp LP) and Review Manager 5 (v. 5.3, Copenhagen, Denmark: Nordic Cochrane Centre). We summarized study and patient characteristics using descriptive statistics. We narratively summarized study design, inclusion criteria, primary outcome(s) and intervention and comparator group content.

We assessed statistical heterogeneity between studies using visual inspection of forest plots, the chi-square test (*α* = 0.10) and the *I*^2^ statistic, using cut-offs established by the Cochrane collaborators (0–40%: might not be important, 30–50%: may represent moderate heterogeneity, 50–90%: may represent substantial heterogeneity, and 75–100%: considerable heterogeneity) [18]. In addition to these cut-offs, we also considered the magnitude of the *I*^2^ statistic (from 0 to 100%) and the *p*-value of the chi-square test when assessing statistical heterogeneity [18]. To supplement statistical assessment, we narratively described clinical and methodological differences.

#### Meta-analyses

We used the Stata command “metaprop” to calculate pooled retention rates [26]. We planned to use random-effects models if there was clinical or methodological heterogeneity and fixed-effects models in the absence of heterogeneity [18]. We used the Freeman-Tukey double arcsine transformation to improve the statistical properties of proportions and to ensure pooled estimates were within 0–100% [26, 27].

#### Primary objective

To determine the retention rates from randomization through primary outcome measurement, we calculated the pooled retention rate and 95% CI across all studies [27]. We conducted four sensitivity analyses (1): to examine individual study effect by removing one study from the model at a time [27] (2), to determine the influence of studies that enrolled adults with pre-frailty by removing these from the meta-analysis [18] (3), to determine the influence of studies that did not report a pre-specified outcome or timepoint and (4) excluding studies with a high risk of bias.

#### Secondary objectives

To identify differences in retention rates between intervention and comparator groups, we calculated the pooled retention rate by group. For RCTs with multiple intervention arms, we included each arm independently. To determine retention rates from randomization through intervention completion, we calculated the pooled retention rate across all studies. We used descriptive statistics (counts, frequencies) to summarize reasons for attrition (at any point during the RCT) and reported strategies to maximize retention.

Post hoc, we categorized reasons for attrition as modifiable (those we hypothesized the research team could change) and non-modifiable reasons (those we hypothesized the research team has little to no control). One reviewer categorized reasons and consulted the review team to ensure agreement. We used the Capability Opportunity Motivation Behaviour system (COM-B) to categorize and better understand modifiable reasons for attrition [28]. The COM-B system was developed to understand factors that influence human behaviour; for an individual to engage in a behaviour, they must have the capability, opportunity and motivation to do so [28]. Each of these three components is interactional and influences the likelihood of an individual engaging in a particular behaviour. Categorization according to the COM-B system may help us understand which of the three influencing factors to target to facilitate behaviour change.

## Results

### Literature search

**Fig. 1:**
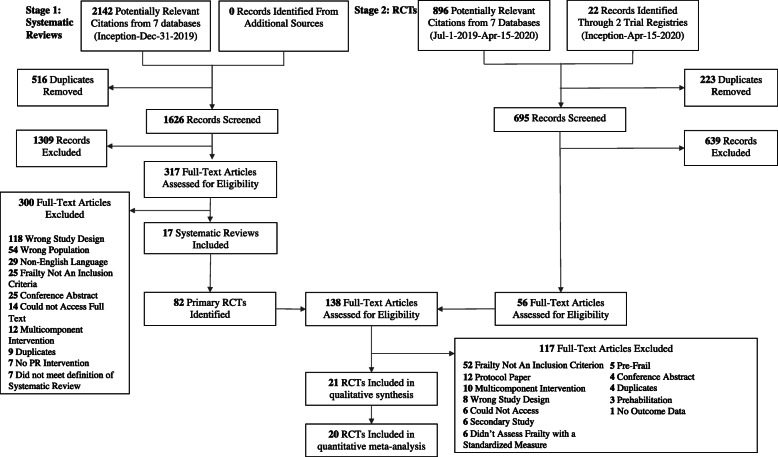
Preferred Reporting Items for Systematic Review and Meta-Analysis (PRISMA) Diagram. Legend: Flow diagram of included studies. We utilized a two-stage search approach, first searching for published systematic reviews of RCTs (left hand side of the diagram), and second searching for primary RCTs (right hand side of the diagram). For systematic reviews that met inclusion, we hand-searched each review to identify the included RCTs. We combined these RCTs at the full-text review

Results are summarized in Fig. 1. In stage 1, we screened 1626 titles and abstracts and 317 full-text articles and identified 17 systematic reviews and 82 primary RCTs within the systematic reviews for further consideration. In stage 1, we had 91.0% reviewer agreement [*κ* = 0.56 (0.43, 0.69)] in title and abstracts and 90.0% agreement [*κ* = 0.46 (0.15, 0.76)] in full-text. In stage 2, we screened 695 unique citations and identified 56 full-texts. Overall, from stages 1 and 2, we reviewed 138 full-text RCTs and included 21 (list of exclusions in Supplementary Table S5). One RCT did not report retention data, thus was only included in the descriptive synthesis of studies. In stage 2, we had 91.0% reviewer agreement [*κ* = 0.46 (0.24, 0.67)] in title and abstracts and 87.0% agreement [*κ* = 0.53 (0.23, 0.83)] in full-text.

### Characteristics of included studies

The 21 included RCTs enrolled 1685 adults with frailty and occurred in 11 countries (Supplementary Table S6, S7). Most RCTs (*n* = 13, 61.9%) applied a parallel-group, two-arm study design. Of 17 RCTs reporting number of centres, most (*n* = 10, 58.8%) were single-centre. Binary outcome data for the cluster RCT were reduced by the design effect (Supplementary Table S8). The median (1st, 3rd quartiles) sample size of RCTs was 76 participants [(46, 90), range = 27–243]. Frailty was measured using 28 assessment tools (13 unique, four studies used more than one [29–33]), with the majority (*n* = 10, 35.7%) using the Fried Frailty Phenotype [2]. Two studies enrolled adults with frailty and pre-frailty [34, 35].

The median proportion of females enrolled was 59.8% [(57.5, 69.8), range = 49–100] and the weighted median participant age was 82.5 years (79.0, 82.2). We identified 24 intervention groups across 21 studies (three studies with two intervention arms [34, 36, 37]). We identified differences in intervention frequency, intensity, type, volume, duration and setting (Supplementary Table S6); however, most studies implemented a multicomponent PR intervention (*n* = 13, 54.2%).

### Methodological quality

**Fig. 2:**
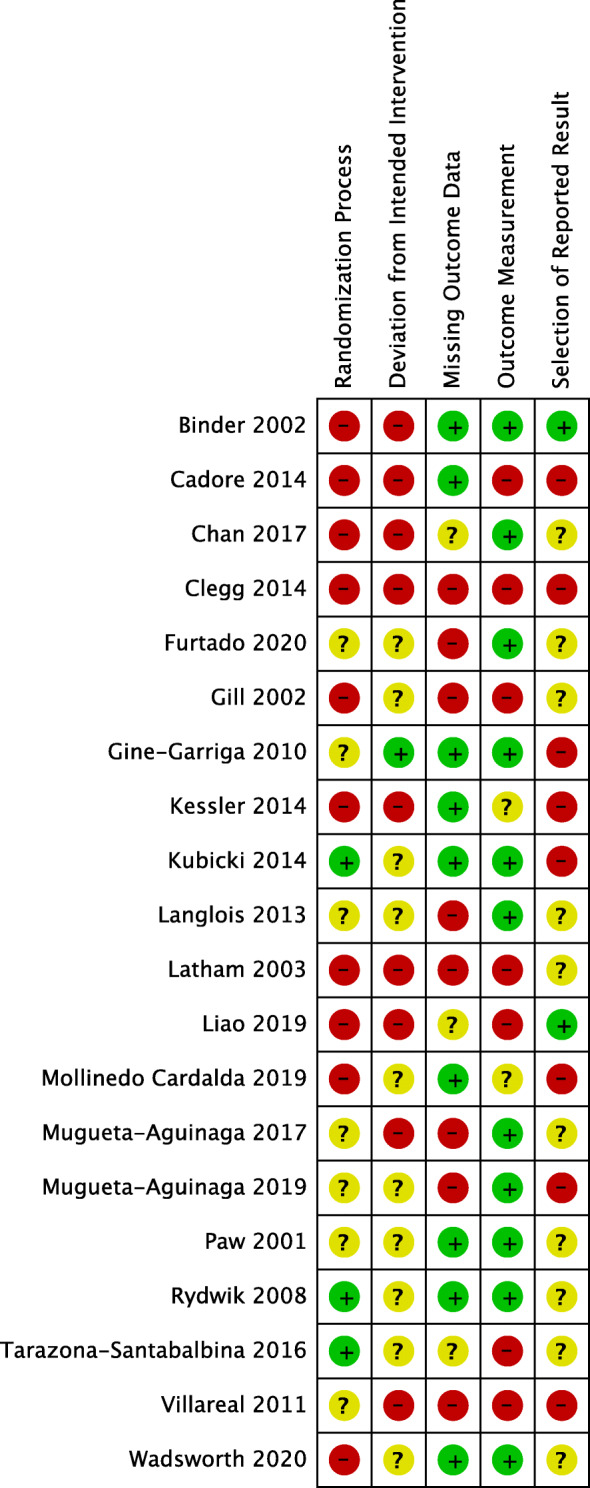
Risk of bias assessments by study. Legend: We assessed risk of bias as high (red circles with “−” ), some concern (yellow circles with “?”) or low (green circles with “+”) or according to the definitions outlined in the Cochrane Handbook [18]. A study was considered to have high overall risk of bias if they had one or more item with a high risk of bias. A study was considered to have some concern overall if they had one or more items with some concern, but no items with high risk of bias. A study was considered to have low overall risk of bias if all items had a low risk of bias

Of 20 studies reporting retention data, all had moderate to high ROB (Fig. 2). High ROB was most frequently present in the randomization process (e.g. notable baseline differences between groups) (*n* = 10, 50.0%) and in deviations from the intended interventions (e.g. participants not analysed as intention-to-treat) (*n* = 9, 45.0%).

### Meta-analysis

Given the presence of clinical and methodological heterogeneity, we used random-effects models for all meta-analyses.

#### Primary outcome

**Fig. 3:**
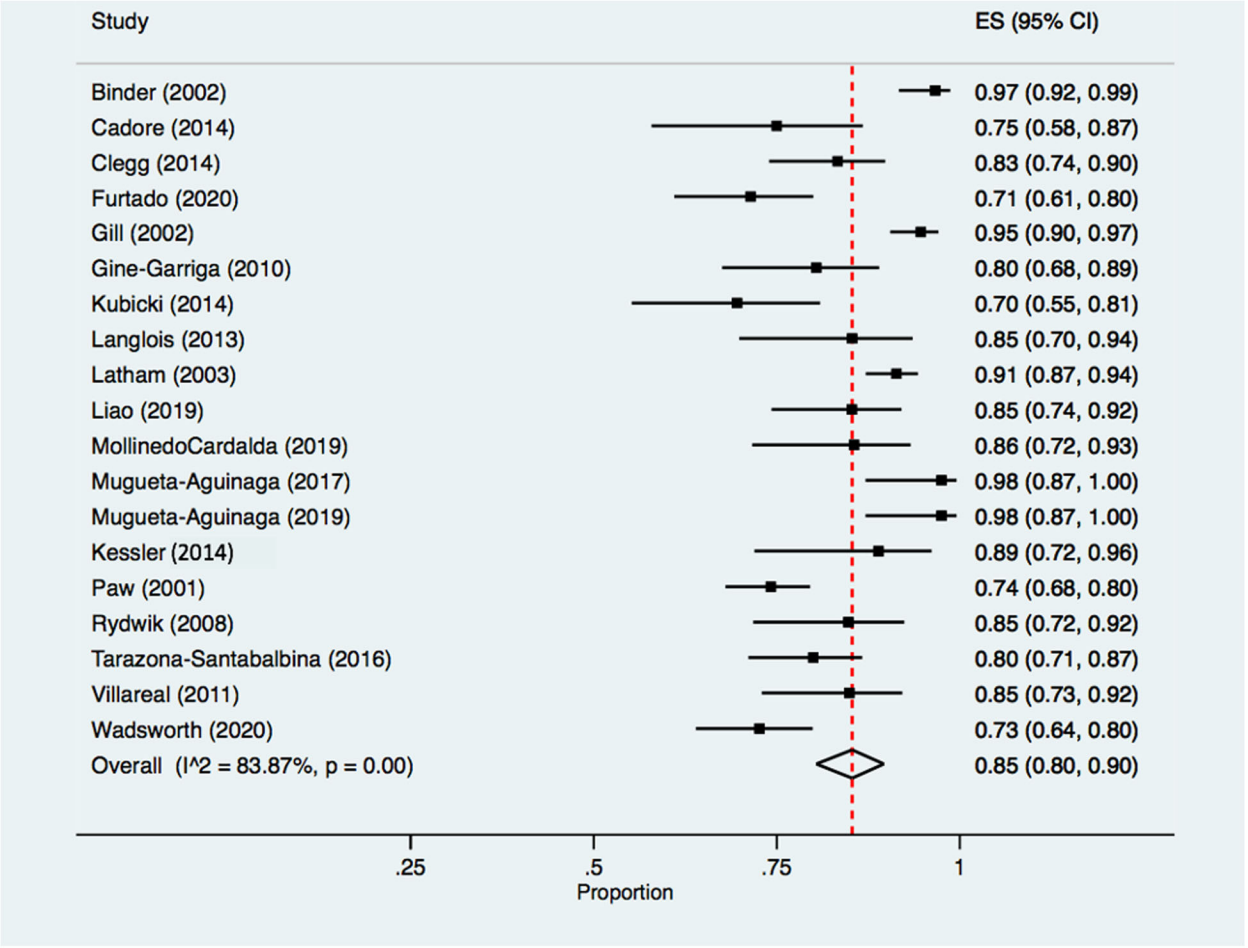
Retention rates from randomization through to primary outcome measurement. Legend: Retention rates and 95% confidence intervals for studies reporting participant retention to primary outcome, as a proportion. Black squares represent point estimates, with accompanying black horizontal lines representing 95% confidence intervals. The diamond and vertical dashed line represent the pooled retention rate. The width of the diamond represents the pooled confidence interval. Heterogeneity Statistics: Tau^2^ = 0.06; chi-square = 116.60, df = 18 (*p* = 0.00). Test for overall effect: *Z* = 34.24 (*p* = 0.00). Abbreviations: ES = effect size

Of 20 studies reporting retention data, 19 reported retention to outcome measurement. Five studies (26.3%) did not specify a primary timepoint [30, 34, 38–40] and three studies (15.8%) did not report a primary outcome [36, 41, 42]. The pooled retention rate across 19 studies was 85.0% [(80.0, 90.0), *I*^2^ = 83.9 (*p* < 0.05), chi-square = 116.60 (*p* < 0.05)] (Fig. 3). The pooled retention rate was robust to the influence of individual studies, to studies that enrolled adults with frailty and pre-frailty, and to studies that did not report a primary outcome or timepoint (Supplementary Figure S1, Figure S2, Table S9). Retention rates were lower when excluding studies with a high risk of bias [76.0% (71.0, 81.0)]; however, only two studies were included in this sensitivity analysis (Supplementary Figure S3).

#### Secondary outcomes

Of 20 studies that reported retention to the primary outcome, 19 reported retention by group, representing 21 intervention groups and 19 comparators. The pooled retention rate for participants in intervention groups was 87.0% [(83.0, 91.0), *I*^2^ = 48.74 (*p* < 0.05), chi-square = 48.74 (*p* < 0.05)], which was not different than comparator groups [85.0% (79.0, 90.0), *I*^2^ = 60.59 (*p* < 0.05), chi-square = 60.59 (*p* < 0.05)].

**Fig. 4:**
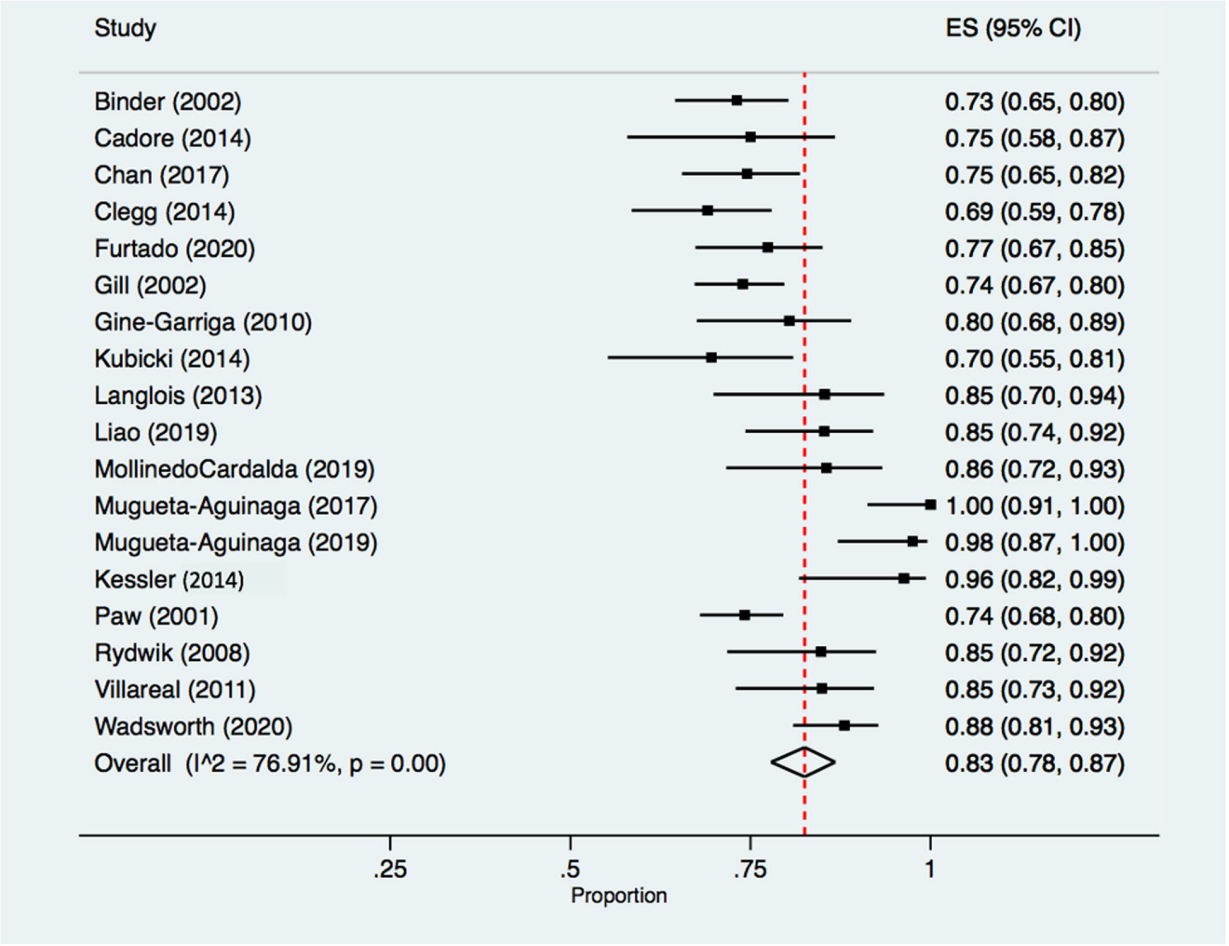
Retention rates from randomization through to intervention completion. Legend: Retention rates and 95% confidence intervals for studies reporting participant retention to intervention completion, as a proportion. Black squares represent point estimates, with accompanying black horizontal lines representing 95% confidence intervals. The diamond and vertical dashed line represent the pooled retention rate. Heterogeneity Statistics: Tau^2^ = 0.04; chi-square = 73.64, df = 17 (*p* = 0.00); *I*^2^ = 76.91%. Test for overall effect: Z = 36.54 (*p* = 0.00). Abbreviations: ES = effect size

Of 20 studies that reported retention data, 18 reported retention to intervention completion, with a pooled rate of 83.0% [(78.0, 87.0), *I*^2^ = 75.91 (*p* < 0.05), chi-square = 73.64 (*p* < 0.05)] (Fig. 4).

Of 20 studies that reported overall study retention, 19 reported reasons for overall attrition and 18 reported the number of participants for each reason (*n* = 347 participants) (Table 1). Studies reported 24 unique reasons for attrition. Fifteen reported non-modifiable reasons for attrition [e.g. participant death (*n* = 12 studies, 63.0%), declining participant health (*n* = 7, 36.8%)] accounting for 48.7% of participants lost to attrition (*n* = 169). Thirteen studies reported potentially modifiable reasons for attrition [e.g. medical reasons related to the study protocol (*n* = 2, 11%). family reasons (*n* = 2, 11%)]. At the participant level, most individuals discontinued participation for reasons related to motivation (*n* = 55 participants, 15.8%). Seventy-eight (22.5%) participants discontinued participation for an unspecified reason, while 19 (5.5%) were lost to follow-up.

**Table 1:** Reported reasons for attrition

Reported reason	Studies, *n* (%) (*N* = 19)^a^	Participants, *n* (%) (*N* = 329)^b^	References
Potentially modifiable reasons			
Capability Pain during exercise Too tired Medical reasons (related to protocol)	4 (21.1) 1 (5.3) 1 (5.3) 2 (10.5)	6 (1.7) 2 (0.6) 1 (0.3) 3 (0.9)	32,40,43,44
Opportunity Family reasons (e.g. family member illness) Too busy Participation requires too much time Job commitments Schedule conflict^b^ Engaged in other activities Enrolled in another study Noncompliance with study protocol	5 (26.3) 2 (10.5) 1 (5.3) 1 (5.3) 1 (5.3) 1 (5.3) 1 (5.3) 1 (5.3) 1 (5.3)	20 (5.8) 2 (0.6) 1 (0.3) 2 (0.6) 1 (0.3) – 11 (0.9) 1 (0.3) 2 (0.6)	29,31,32,38,42
Motivation Lacked interest Low motivation Too much trouble Wanted to lose weight^c^ Physiotherapist perceives rehabilitation needs not met by program	5 (26.3) 2 (10.5) 1 (5.3) 1 (5.3) 1 (5.3) 1 (5.3)	55 (15.9) 9 (2.6) 9 (2.6) 32 (9.2) 1 (0.3) 4 (1.2)	32,33,35,38,43
Discontinued for unspecified reason	7 (36.8)	78 (22.5)	30,31,33,34,39,40,42
Lost to follow-up	3 (15.8)	19 (5.5)	33,38,48
Potentially non-modifiable reasons			
Death Declining health (unrelated to protocol) Hospitalization Surgical intervention preventing participation Participant moved residence Personal reasons (unrelated to protocol)	12 (63.2) 7 (36.8) 3 (15.8) 1 (5.3) 3 (15.8) 2 (10.5)	59 (17.0) 70 (20.2) 20 (5.8) 2 (0.6) 14 (4.0) 4 (1.2)	29–31,33,34,36–41,43,44,50,51

^a^ One study did not report reasons for attrition

^b^ One study did not report reasons for attrition by participant (Langlois 2013)

^c^ Another intervention arm in this study was “weight loss” where participants received a diet intervention

Four studies (20.0%) reported six strategies for maximizing retention [39, 41, 43, 44]: telephone follow-up calls (*n* = 2, 33.3%) [39, 43]; group discussions (*n* = 1, 16.7%) [44]; personal interviews (*n* = 1, 16.7%) [44]; provision of transportation (*n* = 1, 16.7%) [43] and playing music (*n* = 1, 16.7%) [41]. No studies reported evaluation of retention strategies.

## Discussion

### Summary of main results

To our knowledge, this is the first systematic review of participant retention rates in trials of PR for adults with frailty. Twenty of 21 RCTs reported details on participant retention. We identified a pooled retention rate of 85.0% (80.0, 90.0) to primary outcome measurement, with no differences by study group or in retention to intervention completion. Half of the participants who discontinued participation had a potentially modifiable reason which could be addressed in study design or execution. Few studies reported strategies for maximizing retention.

Our results suggest acceptable rates of retention (≥ 85% [9]) according to the PEDro scale for rating RCT quality [9]. Our estimate of retention is higher than the suggested 80% retention cut-off for “high-quality” RCTs of therapeutic interventions described by the Centre for Evidence-Based Medicine [45]. Our pooled participant retention rate is also higher than that anticipated by six RCTs reporting an adjustment for attrition in their sample size calculations [34, 36–39, 42]. Of these six RCTs, the majority (*n* = 5) anticipated a participant retention rate ≤ 80%. Only one RCT reported their rationale for anticipated retention [34]. This rationale was based on a systematic review of exercise programs in older adults where retention rates were between 65 and 75% [46]. However, the rationale for anticipated retention of ≤ 80% in the remaining four studies was not clear. These estimates could be influenced by researchers’ personal experiences of lower retention rates in unpublished trials, emphasizing the importance of trial registries and public reporting of the results of all trials.

### Risk of bias and heterogeneity

Our included studies had a high ROB and heterogeneity. All studies had high or some concern for ROB, potentially resulting in an overestimate of retention rates by treatment group. Half had a high ROB in the randomization process; failures in the conduct or reporting of randomization decreased our confidence that allocation was random, increasing the likelihood of selection bias [47]. If participants were not properly randomized, we cannot be certain differences in participants’ baseline characteristics did not influence retention by group. For example, higher levels of education were associated with decreased attrition in RCTs of weight loss interventions for adults who are overweight or obese [48]. Almost half of the RCTs in our review had a high ROB as a result of deviations from the intended interventions in intervention and control groups. If participants did not receive the intended intervention, we cannot be certain of the generalizability of retention rates to similar interventions applied in research or practice.

We identified statistical heterogeneity in our primary and secondary analyses, with an *I*^2^ value of ≥ 49% for all analyses and a *p*-value < 0.05 for all chi-square tests. Clinically, we documented differences in the frequency (2×/week [36, 38, 40, 41, 43, 49]–5×/week [41, 42]), intensity (e.g. 0 [32]–14 [38] rating of perceived exertion), type (e.g. acupressure [50], strength training [36, 39]), time (10 [44]–90 [32] min), duration (3 [51]–52 [32] weeks) and setting (e.g. residential care/retirement homes [36, 37, 44], primary care centre [38, 42]) of PR interventions. Differences also occurred in the types of comparator groups (e.g. usual care [31, 33, 36, 37, 42, 50–52], PR treatments with different parameters [29, 35, 38, 41, 53]). Despite heterogeneity, we identified narrow CIs (< 11%) for each of our meta-analyses, increasing precision and confidence in our results [18].

### Relationship to previous PR literature

While we were unable to find any systematic reviews that screened for frailty and documented retention, our results are similar to other systematic reviews of retention in RCTs of PR in populations with chronic health conditions (e.g. 82.8% for adults with major depressive disorder [54], 93% for adults with cancer, cardiovascular disease or diabetes [55]) and 90% in people with multimorbidity [56]. In contrast, systematic reviews of PR interventions in adults with human immunodeficiency viruses (*n* = 36 RCTs, 71%) [57] and schizophrenia (*n* = 19 RCTs, 73%) [58] documented lower retention rates. Individuals with frailty may have one or more chronic health conditions; however, the presence of chronic health conditions does not ascertain frailty [59]. Frailty is a multidimensional construct encompassing more than chronic health conditions (e.g. performance of activities of daily living, cognitive performance) [60]. While the concepts of frailty and multimorbidity have been used interchangeably, it is essential that we recognize and study these as distinct clinical syndromes [59].

### Assessment of frailty

We excluded 117 of 138 full-text articles (85%). Half of studies (*n* = 58) were excluded because they either (1) did not identify frailty as an inclusion criterion or (2) did not use a standardized tool or measure to assess frailty. Explicit inclusion/exclusion criteria are necessary to reduce sampling bias and to ensure recruited participants are representative of the population(s) being studied [61]. Screening measures have been validated to rapidly identify frailty (e.g. Gérontopôle Frailty Screening Tool [62], Cardiovascular Health Study Frailty Screening Scale [2, 63], Clinical Frailty Scale [64], Fried Frailty Phenotype [2]) and can be readily implemented into screening for RCT enrollment, ensuring the representativeness of participants and transferability of results.

### Primary outcome reporting

More than one third of RCTs did not specify a primary outcome or timepoint. A priori selection of primary outcomes and timepoints are critical to informing sample size calculations and reducing ROB in the reporting of trial results [65]. Identifying primary outcomes may also enable researchers to prioritize and focus their retention efforts. Practically, non-reporting of primary outcomes and timepoints forces researchers and clinicians to make assumptions when interpreting study results. In the current review, we included the outcomes and timepoints closest to intervention completion which may have led to imprecision.

### Limitations and strengths

Our review has important limitations. First, we did not conduct subgroup analyses to identify potential predictors of attrition. Exploring predictors (e.g. intervention type, setting) may identified specific characteristics of PR that are conducive to higher retention. Second, due to time and resource constraints, we only included studies in English, introducing language bias [18]. Third, we did not search for grey literature, which could enhance the breadth of a systematic review search strategy and reduce the risk of publication bias [18]. However, typically grey literature sources, such as conference abstracts, have strict word counts and it is unlikely that these sources would report the detailed data necessary to support our analyses [66]. To maximize the breadth and efficiency of our search, we conducted a 2-stage search strategy, which leveraged existing systematic reviews in addition to a primary search.

Our review also has important strengths. First, our review adds to previous systematic reviews of study retention in PR trials [54, 57, 58, 67], and can be used to inform the design of future reviews of retention rates in different contexts. Second, we used rigorous methods, including prospective protocol registration, a peer-reviewed (Health Research Librarian) search strategy and duplicate screening, data extraction and ROB assessments. These methods facilitate decreased bias and increased reliability in the systematic review process [18].

### Implications for future research

Despite acceptable rates of retention (≥ 85% [9]), more than half of reported reasons for attrition were potentially modifiable, suggesting opportunities for improvement in study design and conduct. The Behaviour Change Wheel can be used to identify behaviour change interventions according to domains of the COM-B system [28]. For example, reasons for attrition related to participant motivation were most common reason in our review. Motivation can be achieved through interventions such as education, persuasion or incentivization [28]. Future research could identify, tailor and implement strategies for maximizing participant retention using the guiding constructs of the COM-B System and the Behaviour Change Wheel. However, to facilitate this, clear and detailed reporting of reasons for attrition is necessary. In our review, we reported reasons for attrition as closely as possible to the primary data developing an important foundational understanding of reasons for participant attrition. However, some reasons (e.g. too much trouble) could have many explanations (e.g. too much trouble to commute to study site or to participate in protocol) and more detail in reporting future research could further enhance our understanding of participant attrition in this context.

We encourage research teams to report retention rates and strategies used to maximize retention. These details of an RCT are not addressed by reporting guidelines such as CONSORT [68], highlighting an important gap in clinical trial reporting. Thorough and transparent reporting of trial processes and retention strategies may allow future researchers to address unanswered questions related to participant retention such as those identified in the Prioritising Retention in Randomized Trials (PRioRiTy II) study [69].

## Conclusions

Our results suggest that high retention of adults with frailty in PR interventions is feasible, complementing previous research suggesting the effectiveness of PR. Future trials of PR for adults with frailty could benefit from detailed reporting of rigorous methods to decrease ROB throughout the trial process. Our results can be used to inform sample size calculations in future RCTs of PR interventions for adults with frailty; however, given the high ROB of included studies, our estimates of retention should be used conservatively. Accurate estimates of retention may help researchers avoid under- or over-recruitment. Optimization of recruitment and retention will contribute to increased trial efficiency and decreased research waste.

## Supplementary Information

**Additional file 1: Figure S1.** Sensitivity analysis #1. **Figure S2.** Sensitivity analysis #3. **Figure S3.** Sensitivity analysis #4. **Table S1.** PRISMA Checklist. **Table S2.** Inclusion/exclusion criteria and methodological decisions. **Table S3.** Electronic search strategy. **Table S4.** Data extraction points. **Table S5.** Excluded full-text articles and reasons for exclusion. **Table S6.** Summary of included studies. **Table S7.** Summary of study characteristics and retention rates. **Table S8.** Design effect calculations for Mollinedo Cardalda (2019). **Table S9.** Sensitivity analysis #2.

## Data Availability

The datasets used and/or analysed during the current study are available from the corresponding author on reasonable request.

## Abbreviations

PR: Physical rehabilitation
RCT: Randomized controlled trial
CI: Confidence interval
PEDro: Physiotherapy Evidence Database
PRISMA: Preferred Reporting Items for Systematic Review and Meta-Analysis
CINAHL: The Cumulative Index to Nursing and Allied Health Literature
AMED: Allied and Complementary Medicine Database
ROB: Risk of bias
COM-B: Capability Opportunity Motivation Behaviour System

## References

[1] Clegg A, Young J, Iliffe S, Rikkert MO, Rockwood K (2013). Frailty in elderly people. Lancet.

[2] Fried LP, Tangen CM, Walston J, Newman AB, Hirsch C, Gottdiener J, Seeman T, Tracy R, Kop WJ, Burke G, McBurnie MA (2001). Frailty in older adults: evidence for a phenotype. J Gerontol A Biol Sci Med Sci.

[3] O’Hoski S, Bean JF, Ma J, So HY, Kuspinar A, Richardson J, Wald J, Beauchamp MK (2020). Physical function and frailty for predicting adverse outcomes in older primary care patients. Arch Phys Medicine and Rehabil.

[4] Langlois F, Vu TTM, Kergoat MJ, Chassé K, Dupuis G, Bherer L (2012). The multiple dimensions of frailty: physical capacity, cognition, and quality of life. Int Psychogeriatr.

[5] Iecovich E (2008). Caregiving burden, community services, and quality of life of primary caregivers of frail elderly persons. J Appl Gerontol.

[6] Canadian Fraily Network. Annual Report 2016-2017. Frailty Matters: Every Canadian is affected by frailty. 2017. https://www.cfn-nce.ca/wp-content/uploads/2018/09/cfn-2016-17-annual-report.pdf.

[7] Zhang Y, Zhang Y, Du S, Wang Q, Xia H, Sun R (2019). Exercise interventions for improving physical function, daily living activities and quality of life in community-dwelling frail older adults: a systematic review and meta-analysis of randomized controlled trials. Geriatr Nurs.

[8] Chou C-H, Hwang C-L, Wu Y-T (2012). Effect of exercise on physical function, daily living activities, and quality of life in the frail older adults: a meta-analysis. Arch Phys Med Rehabil.

[9] Maher CG, Sherrington C, Herbert RD, Moseley AM, Elkins M (2003). Reliability of the PEDro Scale for rating quality of randomized controlled trials. Phys Ther.

[10] Kaur M, Sprague S, Ignacy T, Thoma A, Bhandari M, Farrokhyar F (2014). Practical tips for surgical research. Can J Surg.

[11] Page SJ, Persch AC (2013). Recruitment, retention, and blinding in clinical trials. Am J Occup Ther.

[12] O’Grady HK, Farley C, Takaoka A, Mayens E, Kho M. Participant retention rates in randomized clinical trials of physical rehabilitation for adults with frailty: A systematic review and meta-analysis. 2020. Retrieved from osf.io/jm7hu. 10.1186/s13063-022-06172-5 PMC8961921 35346320

[13] Moher D (2009). Preferred Reporting Items for Systematic Reviews and Meta-Analyses: the PRISMA Statement. Ann Intern Med.

[14] World Health Organization. Rehabilitation 2030: A call for action. Geneva: World Health Organization. 2017.

[15] Ferreira V, Agnihotram RV, Bergdahl A, van Rooijen SJ, Awasthi R, Carli F, Scheede-Bergdahl C (2018). Maximizing patient adherence to prehabilitation: what do the patients say?. Support Care Cancer.

[16] Carli F, Zavorsky GS (2005). Optimizing functional exercise capacity in the elderly surgical population. Curr Opin Clin Nutr Metab Care.

[17] Lee E, Dobbins M, DeCorby K, McRae L, Tirilis D, Husson H (2012). An optimal search filter for retrieving systematic reviews and meta-analyses. BMC Med Res Methodol.

[18] Higgins JPT, Thomas J, Chandler J, Cumpston M, Li T, Page MJ, Welch VA (editors). Cochrane Handbook for Systematic Reviews of Interventions version 6.3 (updated February 2022). Cochrane. 2022. Available from www.training.cochrane.org/handbook.

[19] University of Alberta Libraries. Subject guides: Systematic Reviews, Scoping Reviews, and Health Technology Assessments: Searching the Literature. University of Alberta. 2020. Available at: https://guides.library.ualberta.ca/c.php?g=248586&p=1655962.

[20] McHugh ML (2012). Interrater reliability: the kappa statistic. Biochem Med (Zagreb).

[21] Sterne JAC, Savović J, Page MJ, Elbers RG, Blencowe NS, Boutron I, Cates CJ, Cheng HY, Corbett MS, Eldridge SM, Emberson JR, Hernán MA, Hopewell S, Hróbjartsson A, Junqueira DR, Jüni P, Kirkham JJ, Lasserson T, Li T, McAleenan A, Reeves BC, Shepperd S, Shrier I, Stewart LA, Tilling K, White IR, Whiting PF, Higgins JPT (2019). RoB 2: a revised tool for assessing risk of bias in randomised trials. BMJ.

[22] Liberati A, Altman DG, Tetzlaff J, Mulrow C, Gøtzsche PC, Ioannidis JPA, Clarke M, Devereaux PJ, Kleijnen J, Moher D (2009). The PRISMA statement for reporting systematic reviews and meta-analyses of studies that evaluate health care interventions: explanation and elaboration. J Clin Epidemiol.

[23] Brueton VC, Tierney JF, Stenning S, Meredith S, Harding S, Nazareth I, Rait G (2014). Strategies to improve retention in randomised trials: a Cochrane systematic review and meta-analysis. BMJ Open.

[24] Slade SC, Dionne CE, Underwood M, Buchbinder R (2016). Consensus on Exercise Reporting Template (CERT): explanation and elaboration statement. Br J Sports Med.

[25] Rao JNK, Scott AJ (1992). A simple method for the analysis of clustered binary data. Biometrics.

[26] Nyaga VN, Arbyn M, Aerts M (2014). Metaprop: a Stata command to perform meta-analysis of binomial data. Arch Public Health.

[27] Munn Z, Moola S, Lisy K, Riitano D, Tufanaru C. Chapter 5: Systematic reviews of prevalence and incidence. In: Aromataris E, Munn Z (Editors). JBI Manual for Evidence Synthesis. 4th ed. JBI; 2019. Available from: https://synthesismanual.jbi.global. 10.46658/JBIRM-17-05.

[28] Michie S, van Stralen MM, West R (2011). The behaviour change wheel: a new method for characterising and designing behaviour change interventions. Implement Sci.

[29] Binder EF, Schechtman KB, Ehsani AA, Steger-May K, Brown M, Sinacore DR, Yarasheski KE, Holloszy JO (2002). Effects of exercise training on frailty in community-dwelling older adults: results of a randomized, controlled trial. J Am Geriatr Soc.

[30] Gill T, Baker D, Gottschalk M, Peduzzi P, Allore H, Byers A (2002). A program to prevent functional decline in physically frail, elderly persons who live at home. N Engl J Med.

[31] Langlois F, Vu TTM, Chassé K, Dupuis G, Kergoat MJ, Bherer L (2013). Benefits of physical exercise training on cognition and quality of life in frail older adults. J Gerontol B Psychol Sci Soc Sci.

[32] Villareal DT, Chode S, Parimi N, Sinacore DR, Hilton T, Armamento-Villareal R, Napoli N, Qualls C, Shah K (2011). Weight loss, exercise, or both and physical function in obese older adults. N Engl J Med.

[33] Clegg A, Barber S, Young J, Iliffe S, Forster A (2014). The Home-based Older People’s Exercise (HOPE) trial: a pilot randomised controlled trial of a home-based exercise intervention for older people with frailty. Age Ageing.

[34] Furtado GE, Carvalho HM, Loureiro M, Patrício M, Uba-Chupel M, Colado JC, Hogervorst E, Ferreira JP, Teixeira AM (2020). Chair-based exercise programs in institutionalized older women: salivary steroid hormones, disabilities and frailty changes. Exp Gerontol.

[35] Liao YY, Chen IH, Wang RY (2019). Effects of Kinect-based exergaming on frailty status and physical performance in prefrail and frail elderly: a randomized controlled trial. Sci Rep.

[36] Mollinedo Cardalda I, Lopez A, Cancela Carral JM (2019). The effects of different types of physical exercise on physical and cognitive function in frail institutionalized older adults with mild to moderate cognitive impairment. A randomized controlled trial. Arch Gerontol Geriatr.

[37] Wadsworth D, Lark S (2020). Effects of whole-body vibration training on the physical function of the frail elderly: an open, randomized controlled trial. Arch Phys Med Rehabil.

[38] Gine-Garriga M, Guerra M, Pages E, Manini TM, Jimenez R, Unnithan VB (2010). The effect of functional circuit training on physical frailty in frail older adults: a randomized controlled trial. J Aging Phys Act.

[39] Latham N, Anderson C, Lee A (2003). A randomized, controlled trial of quadriceps resistance exercise and vitamin D in frail older people: The Frailty Interventions Trial in Elderly Subjects (FITNESS). J Am Geriatr Soc.

[40] Rydwik E, Lammes E, Frandin K, Akner G (2008). Effects of a physical and nutritional intervention program for frail elderly people over age 75. A randomized controlled pilot treatment trial. Aging Clin Exp Res.

[41] Cadore EL, Casas-Herrero A, Zambom-Ferraresi F, Idoate F, Millor N, Gómez M, Rodriguez-Mañas L, Izquierdo M (2014). Multicomponent exercises including muscle power training enhance muscle mass, power output, and functional outcomes in institutionalized frail nonagenarians. Age.

[42] Tarazona-Santabalbina FJ, Gomez-Cabrera MC, Perez-Ros P (2016). A multicomponent exercise intervention that reverses frailty and improves cognition, emotion, and social networking in the community-dwelling frail elderly: a randomized clinical trial. J Am Med Dir Assoc.

[43] Paw M, de Jong N, Schouten E, Hiddink G, Kok F (2001). Physical exercise and/or enriched foods for functional improvement in frail, independently living elderly: a randomized controlled trial. Arch Phys Med Rehabil.

[44] Kessler J, Radlinger L, Baur H, Rogan S (2014). Effect of stochastic resonance whole body vibration on functional performance in the frail elderly: A pilot study. Arch Gerontol Geriatr.

[45] Burns PB, Rohrich RJ, Chung KC (2011). The levels of evidence and their role in evidence-based medicine. Plast Reconstr Surg.

[46] Picorelli AMA, Pereira LSM, Pereira DS, Felício D, Sherrington C (2014). Adherence to exercise programs for older people is influenced by program characteristics and personal factors: a systematic review. J Physiother.

[47] Sterne JAC, Savović J, Page MJ, Elbers RG, Blencowe NS, Boutron I, Cates CJ, Cheng H-Y, Corbett MS, Eldridge SM, Hernán MA, Hopewell S, Hróbjartsson A, Junqueira DR, Jüni P, Kirkham JJ, Lasserson T, Li T, McAleenan A, Reeves BC, Shepperd S, Shrier I, Stewart LA, Tilling K, White IR, Whiting PF, Higgins JPT. RoB 2: a revised tool for assessing risk of bias in randomised trials. BMJ 2019;366:l4898. 10.1136/bmj.l4898 31462531

[48] Moroshko I, Brennan L, O’Brien P (2011). Predictors of dropout in weight loss interventions: a systematic review of the literature. Obes Rev.

[49] Kubicki A, Bonnetblanc F, Petrement G, Mourey F (2014). Motor-prediction improvements after virtual rehabilitation in geriatrics: frail patients reveal different learning curves for movement and postural control. Neurophysiologie Clinique/Clin Neurophysiol.

[50] Chan CWC, Chau PH, Leung AYM, Lo KC, Shi H, Yum TP, Lee YY, Li L (2017). Acupressure for frail older people in community dwellings—a randomised controlled trial. Age Ageing.

[51] Mugueta-Aguinaga I, Garcia-Zapirain B. FRED: Exergame to prevent dependence and functional deterioration associated with ageing. A pilot three-week randomized controlled clinical trial. Int J Environ Res Public Health. 2017;14(12):1439. 10.3390/ijerph14121439. 10.3390/ijerph14121439 PMC5750858 29168787

[52] Mugueta-Aguinaga I, Garcia-Zapirain B. Frailty level monitoring and analysis after a pilot six-week randomized controlled clinical trial using the FRED Exergame Including Biofeedback Supervision in an Elderly Day Care Centre. Int J Environ Res Public Health. 2019;16(5):729. 10.3390/ijerph16050729. 10.3390/ijerph16050729 PMC6427585 30823460

[53] Brown M, Sinacore DR, Ehsani AA, Binder EF, Holloszy JO, Kohrt WM (2000). Low-intensity exercise as a modifier of physical frailty in older adults. Arch Phys Med Rehabil.

[54] Stubbs B, Vancampfort D, Rosenbaum S, Ward PB, Richards J, Soundy A, Veronese N, Solmi M, Schuch FB (2016). Dropout from exercise randomized controlled trials among people with depression: a meta-analysis and meta regression. J Affect Disord.

[55] Bullard T, Ji M, An R, Trinh L, Mackenzie M, Mullen SP (2019). A systematic review and meta-analysis of adherence to physical activity interventions among three chronic conditions: cancer, cardiovascular disease, and diabetes. BMC Public Health.

[56] Harris LK, Skou ST, Juhl CB, Jäger M, Bricca A (2021). Recruitment and retention rates in randomised controlled trials of exercise therapy in people with multimorbidity: a systematic review and meta-analysis. Trials.

[57] Vancampfort D, Mugisha J, Richards J, de Hert M, Lazzarotto AR, Schuch FB, Probst M, Stubbs B (2017). Dropout from physical activity interventions in people living with HIV: a systematic review and meta-analysis. AIDS Care.

[58] Vancampfort D, Rosenbaum S, Schuch FB, Ward PB, Probst M, Stubbs B (2016). Prevalence and predictors of treatment dropout from physical activity interventions in schizophrenia: a meta-analysis. Gen Hospital Psychiatr.

[59] Morley JE, Vellas B (2013). Abellan van Kan G, et al. Frailty consensus: a call to action. J Am Med Dir Assoc.

[60] Searle SD, Mitnitski A, Gahbauer EA, Gill TM, Rockwood K (2008). A standard procedure for creating a frailty index. BMC Geriatr.

[61] Kendall J (2003). Designing a research project: randomised controlled trials and their principles. Emerg Med J.

[62] Subra J, Gillette-Guyonnet S, Cesari M, Oustric S, Vellas B (2012). The Platform Team. The integration of frailty into clinical practice: preliminary results from the Gérontopôle. J Nutr Health Aging.

[63] Walston J, McBurnie MA, Newman A, Tracy RP, Kop WJ, Hirsch CH, Gottdiener J, Fried LP, Cardiovascular Health Study (2002). Frailty and activation of the inflammation and coagulation systems with and without clinical comorbidities: results from the cardiovascular health study. Arch Intern Med.

[64] Rockwood K, Song X, MacKnight C, Bergman H, Hogan DB, McDowell I, Mitnitski A (2005). A global clinical measure of fitness and frailty in elderly people. CMAJ.

[65] Andrade C (2015). The primary outcome measure and its importance in clinical trials. J Clin Psychiatry.

[66] Mahood Q, Eerd DV, Irvin E (2014). Searching for grey literature for systematic reviews: challenges and benefits. Res Synth Methods.

[67] Cramer H, Haller H, Dobos G, Lauche R. A systematic review and meta-analysis estimating the expected dropout rates in randomized controlled trials on yoga interventions. Evid Based Complement Altern Med. 2016;2016:7. 10.1155/2016/5859729. 10.1155/2016/5859729 PMC4927989 27413387

[68] Schulz KF, Altman DG, Moher D, the CONSORT Group (2010). CONSORT 2010 Statement: updated guidelines for reporting parallel group randomised trials. BMC Med.

[69] Brunsdon D, Biesty L, Brocklehurst P, Brueton V, Devane D, Elliott J, Galvin S, Gamble C, Gardner H, Healy P, Hood K, Jordan J, Lanz D, Maeso B, Roberts A, Skene I, Soulsby I, Stewart D, Torgerson D, Treweek S, Whiting C, Wren S, Worrall A, Gillies K (2019). What are the most important unanswered research questions in trial retention? A James Lind Alliance Priority Setting Partnership: the PRioRiTy II (Prioritising Retention in Randomised Trials) study. Trials.

