# Supplementary material for: Retention in RCTs of physical rehabilitation for adults with frailty: a systematic review and meta-analysis
Source: Trials. 2022 Mar 28;23:235. doi: 10.1186/s13063-022-06172-5 (PMC8961921; doi:10.1186/s13063-022-06172-5)
Supplement: Supplementary file 1 — Additional file 1: Figure S1. Sensitivity analysis #1. Figure S2. Sensitivity analysis #3. Figure S3. Sensitivity analysis #4. Table S1. PRISMA Checklist. Table S2. Inclusion/exclusion criteria and methodological decisions. Table S3. Electronic search strategy. Table S4. Data extraction points. Table S5. Excluded full-text articles and reasons for exclusion. Table S6. Summary of included studies. Table S7. Summary of study characteristics and retention rates. Table S8. Design effect calculations for Mollinedo Cardalda (2019). Table S9. Sensitivity analysis #2. [file 13063_2022_6172_MOESM1_ESM.docx]

**Supplementary Data File**

**Table of Contents Page**

Supplementary Figure S1. Sensitivity analysis #1. 2

Supplementary Figure S2. Sensitivity analysis #3. 3

Supplementary Figure S3. Sensitivity analysis #4. 4

Supplementary Table S1. PRISMA checklist. 5

Supplementary Table S2. Inclusion/exclusion criteria and methodological decisions. 7

Supplementary Table S3. Electronic search strategy. 8

Supplementary Table S4. Data extraction points. 12

Supplementary Table S5. Excluded full-text articles and reasons for exclusion. 13

Supplementary Table S6. Summary of included studies. 21

Supplementary Table S7. Summary of study characteristics and retention rates. 30

Supplementary Table S8. Design effect calculations for Mollinedo Cardalda (2019). 33

Supplementary Table S9. Sensitivity analysis #2. 34

**Supplementary Figure S1.** Retention rates from randomization through to primary outcome measurement excluding studies that enrolled adults with frailty and pre-frailty (Furtado (2020), Liao (2019)).


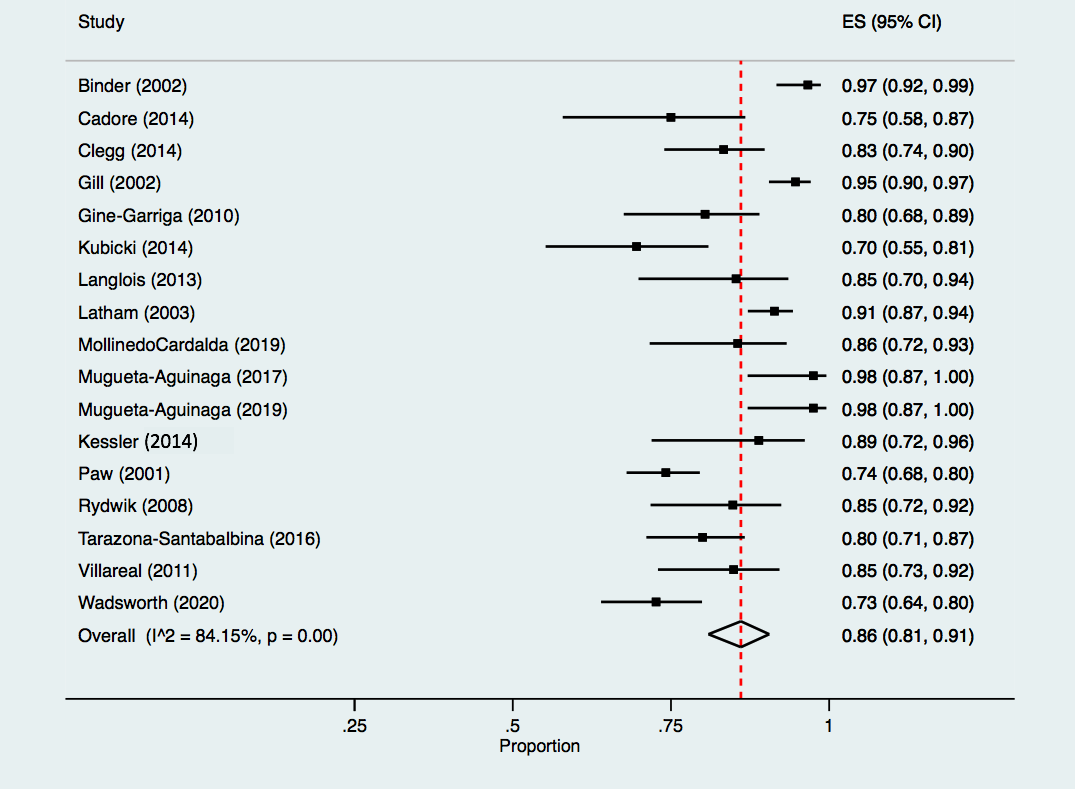


Legend: Retention rates and 95% confidence intervals, omitting studies that enrolled adults with frailty and pre-frailty, as a proportion. Black squares represent point estimates, with accompanying black horizontal lines representing 95% confidence intervals. The diamond and vertical red dashed line represent the pooled retention rate. The width of the black diamond represents the pooled confidence interval.

Heterogeneity Statistics: Tau^2^ = 0.06; Chi^2^ = 100.93, df = 16 (p=0.00); I^2^ = 84.15%

Test for overall effect: Z = 32.48 (p=0.00)

Abbreviations: ES = Effect Size

**Supplementary Figure S2.** Retention rates from randomization through to primary outcome measurement, excluding studies that did not report a primary outcome or timepoint.

**
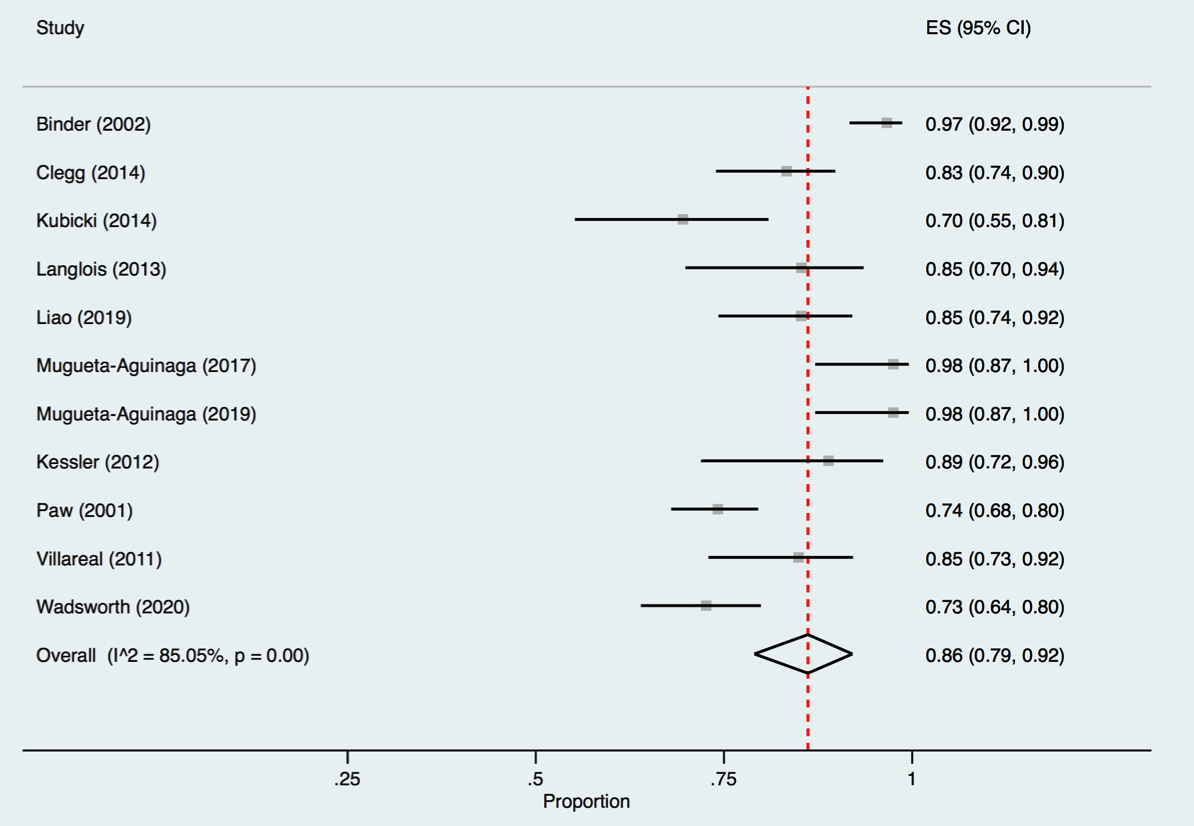
**

Legend: Retention rates and 95% confidence intervals, omitting studies that enrolled adults with frailty and pre-frailty, as a proportion. Black squares represent point estimates, with accompanying black horizontal lines representing 95% confidence intervals. The diamond and vertical red dashed line represent the pooled retention rate. The width of the black diamond represents the pooled confidence interval.

Heterogeneity Statistics: Tau^2^ = 0.08; Chi^2^ = 66.88, df = 10 (p=0.00); I^2^ = 85.05%

Test for overall effect: Z = 23.84 (p=0.00)

Abbreviations: ES = Effect Size

**Supplementary Figure S3.** Retention rates from randomization through to primary outcome measurement, excluding studies with a high overall risk of bias.

Legend: Retention rates and 95% confidence intervals, omitting studies that enrolled adults with frailty and pre-frailty, as a proportion. Black squares represent point estimates, with accompanying black horizontal lines representing 95% confidence intervals. The diamond and vertical red dashed line represent the pooled retention rate. The width of the black diamond represents the pooled confidence interval.

Test for overall effect: Z = 32.54 (p=0.00)

Abbreviations: ES = Effect Size

**Supplementary Table S1.** Preferred Reporting Items for Systematic Review and Meta-Analysis (PRISMA) checklist.

| **Section/topic** | **#** | **Checklist item** | **Reported on page #** |
| --- | --- | --- | --- |
| **TITLE** | | |  |
| Title | 1 | Identify the report as a systematic review, meta-analysis, or both. | 1 |
| **ABSTRACT** | | |  |
| Structured summary | 2 | Provide a structured summary including, as applicable: background; objectives; data sources; study eligibility criteria, participants, and interventions; study appraisal and synthesis methods; results; limitations; conclusions and implications of key findings; systematic review registration number. | 2-3 |
| **INTRODUCTION** | | |  |
| Rationale | 3 | Describe the rationale for the review in the context of what is already known. | 4-5 |
| Objectives | 4 | Provide an explicit statement of questions being addressed with reference to participants, interventions, comparisons, outcomes, and study design (PICOS). | 5 |
| **METHODS** | | |  |
| Protocol and registration | 5 | Indicate if a review protocol exists, if and where it can be accessed (e.g., Web address), and, if available, provide registration information including registration number. | 5 |
| Eligibility criteria | 6 | Specify study characteristics (e.g., PICOS, length of follow-up) and report characteristics (e.g., years considered, language, publication status) used as criteria for eligibility, giving rationale. | 5-6 |
| Information sources | 7 | Describe all information sources (e.g., databases with dates of coverage, contact with study authors to identify additional studies) in the search and date last searched. | 6 |
| Search | 8 | Present full electronic search strategy for at least one database, including any limits used, such that it could be repeated. | Supplementary Table 3 |
| Study selection | 9 | State the process for selecting studies (i.e., screening, eligibility, included in systematic review, and, if applicable, included in the meta-analysis). | 8 |
| Data collection process | 10 | Describe method of data extraction from reports (e.g., piloted forms, independently, in duplicate) and any processes for obtaining and confirming data from investigators. | 8 |
| Data items | 11 | List and define all variables for which data were sought (e.g., PICOS, funding sources) and any assumptions and simplifications made. | Supplementary Table 4 |
| Risk of bias in individual studies | 12 | Describe methods used for assessing risk of bias of individual studies (including specification of whether this was done at the study or outcome level), and how this information is to be used in any data synthesis. | 9 |
| Summary measures | 13 | State the principal summary measures (e.g., risk ratio, difference in means). | 9-10 |
| Synthesis of results | 14 | Describe the methods of handling data and combining results of studies, if done, including measures of consistency (e.g., I^2^) for each meta-analysis. | 9 |
| Risk of bias across studies | 15 | Specify any assessment of risk of bias that may affect the cumulative evidence (e.g., publication bias, selective reporting within studies). | 9 |
| Additional analyses | 16 | Describe methods of additional analyses (e.g., sensitivity or subgroup analyses, meta-regression), if done, indicating which were pre-specified. | 10 |
| **RESULTS** | | | |
| Study selection | 17 | Give numbers of studies screened, assessed for eligibility, and included in the review, with reasons for exclusions at each stage, ideally with a flow diagram. | 10-11 |
| Study characteristics | 18 | For each study, present characteristics for which data were extracted (e.g., study size, PICOS, follow-up period) and provide the citations. | 11-12 |
| Risk of bias within studies | 19 | Present data on risk of bias of each study and, if available, any outcome level assessment (see item 12). | 12 |
| Results of individual studies | 20 | For all outcomes considered (benefits or harms), present, for each study: (a) simple summary data for each intervention group (b) effect estimates and confidence intervals, ideally with a forest plot. | Table 1 |
| Synthesis of results | 21 | Present results of each meta-analysis done, including confidence intervals and measures of consistency. | 12-13 |
| Risk of bias across studies | 22 | Present results of any assessment of risk of bias across studies (see Item 15). | 12 |
| Additional analysis | 23 | Give results of additional analyses, if done (e.g., sensitivity or subgroup analyses, meta-regression [see Item 16]). | 12-13 |
| **DISCUSSION** | | | |
| Summary of evidence | 24 | Summarize the main findings including the strength of evidence for each main outcome; consider their relevance to key groups (e.g., healthcare providers, users, and policy makers). | 14-16 |
| Limitations | 25 | Discuss limitations at study and outcome level (e.g., risk of bias), and at review-level (e.g., incomplete retrieval of identified research, reporting bias). | 17 |
| Conclusions | 26 | Provide a general interpretation of the results in the context of other evidence, and implications for future research. | 19 |
| **FUNDING** | | | |
| Funding | 27 | Describe sources of funding for the systematic review and other support (e.g., supply of data); role of funders for the systematic review. | 20 |

**Supplementary Table S2.** Inclusion/exclusion criteria and methodological decisions.

| Inclusion Criteria | Decisions |
| --- | --- |
| Adults > 18 years with frailty | - Studies must have explicitly identified frailty as an inclusion criterion and used a standardized tool or measure to assess frailty - We included studies that enrolled adults with frailty and pre-frailty if the majority (>50%) were frail or if results were presented for frail and pre-frail independently (in this case, we included only the participants with frailty in our analysis) |
| Physical rehabilitation interventions | - To identify PR interventions, we reviewed the intervention description and study aims - If authors did not state the aim, we relied on the judgement of the review team (2 Physiotherapists (EM, CF), and 1 Rehabilitation Science PhD Student) to determine whether an intervention was likely to enhance or restore physical function |
| Retention Rate | - If studies did not report a flow diagram of participant enrollment, group allocation, follow-up status and participants included in analysis, we tried to gather this information from text, supplementary data or by contacting authors |

**Supplementary Table S3.** Electronic search strategy developed in consultation with a health research librarian.

| **Database: OVID Medline** | | |
| --- | --- | --- |
| *Concept* | *Subject Headings* | *Key Words* |
| Adults with Frailty | Frail Elderly/  Frailty/ | frail* |
| Physical Rehabilitation | exp Rehabilitation/  exp Physical Therapy Modalities/ | Rehab*  Physiotherap*  “physical therapy”  Exercise |
| Systematic Review^a^ | Meta Analysis/  Exp Review Literature/ | MEDLINE.tw.  Systematic review.tw.  Meta analysis.pt.  Intervention*.ti. |
| Randomized Controlled Trial^b^ | Exp Randomized Controlled Trial/ | Randomized controlled trial.pt.  Controlled clinical trial.pt.  Random*.ti.ab.  Trial*.ti.ab.  Group*.ti.ab. |
| **Database: EMBASE** | | |
| *Concept* | *Subject Headings* | *Key Words* |
| Adults with Frailty | Frail Elderly/  Frailty/ | Frail* |
| Physical Rehabilitation | exp Rehabilitation/  exp Exercise/  exp Physiotherapy/ | Rehab*  Physiotherap*  “Physical therapy”  Exercise |
| Systematic Review^a^ | Systematic review/  Meta-analysis/ | MEDLINE.tw.  Systematic review.tw  Intervention*.ti. |
| Randomized Controlled Trial^b^ | Exp clinical trial/ | Random*.ti.ab.  Trial*.ti.ab.  Group*.ti.ab. |
| **Database: Ageline** | | |
| *Concept* | *Subject Headings* | *Key Words* |
| Adults with Frailty | Exp Frail elderly/ | Frail* |
| Physical Rehabilitation | Exp Rehabilitation/  Exp Exercise/  Exp Physical Therapy/ | Rehab*  Physiotherap*  “Physical therapy”  Exercise |
| Systematic Review^a^ | † | TI systematic review  AB systematic review  TI meta analysis  AB meta analysis |
| Randomized Controlled Trial^b^ | AB Randomized Controlled Trials/  AB Controlled Clinical Trials/ | Random*.ti.ab.  Trial*.ti.ab.  Group*.ti.ab. |
| **Database: Web of Science** | | |
| *Concept* | *Subject Headings* | *Key Words* |
| Adults with Frailty | ‡ | Frail* |
| Physical Rehabilitation | ‡ | Rehab*  Physiotherap*  “Physical Therapy”  Exercise |
| Systematic Review^a^ | ‡ | Systematic Review  Meta analysis |
| Randomized Controlled Trial^b^ | ‡ | Clinical trial*  Research design*  Comparative stud*  Evaluation stud*  Controlled trial*  Follow-up stud*  Prospective stud*  Random*  Placebo*  Single blind*  Double blind* |
| **Database: Cochrane Library** | | |
| *Concept* | *Subject Headings* | *Key Words* |
| Adults with Frailty | ‡ | Frail* |
| Physical Rehabilitation | ‡ | Rehab* OR  Physiotherap*  "Physical Therapy"  Exercise |
| Systematic Review^a^ | ‡ | N/A |
| Randomized Controlled Trial^b^ | ‡ | "Randomized controlled trial"  "Clinical trial"  Random*  Trial*  Group* |
| **Database: CINAHL** | | |
| *Concept* | *Subject Headings* | *Key Words* |
| Adults with Frailty | Frail elderly/ | Frail* |
| Physical Rehabilitation | Exp Rehabilitation/  Exp Exercise/  Exp Physical Therapy/ | Rehab*  Physiotherap*  “Physical therapy”  Exercise |
| Systematic Review^a^ | Exp systematic review/  Meta analysis/ | TI systematic review  AB systematic review  TI meta analysis  AB meta analysis |
| Randomized Controlled Trial^b^ | TI, AB Randomized Controlled Trials/  TI, AB Controlled Clinical Trials/ | Random*.ti.ab.  Trial*.ti.ab.  Group*.ti.ab. |
| **Database: AMED** | | |
| *Concept* | *Subject Headings* | *Key Words* |
| Adults with Frailty | Frail Elderly/ | frail* |
| Physical Rehabilitation | Exp Rehabilitation/  Exp exercise/  Exp Physiotherapists/  Exp Physical therapy specialty/  Exp Physical therapy modalities/ | Rehab*  Physiotherap*  “physical therapy”  Exercise |
| Systematic Review^a^ | Meta Analysis/ | Meta analysis.pt.  Systematic Review.ti,ab.  Meta Analysis.ti,ab. |
| Randomized Controlled Trial^b^ | Exp Clinical trials/  Exp Randomized Controlled trials/ | Randomized controlled trial.pt.  Clinical trial.pt.  Random*.ti.ab.  Trial*.ti.ab.  Group*.ti.ab. |

**†** Denotes databases without subject headings. **‡** Denotes databases without subject headings.

**^a^** During stage 1 of our literature search, we searched for the concepts “adults with frailty”, “physical rehabilitation” and “systematic review”.

**^b^** During stage 2 of our literature search, we searched for the concepts “adults with frailty”, “physical rehabilitation” and “randomized controlled trial”.

Boolean Operators:

1. We combined all subject headings for each concept, where applicable, using the OR operator.
2. We combined all key words for each concept using the OR operator.
3. Concepts were combined using the AND operator.

**Supplementary Table S4.** Data extraction points.

| Study Element | Data Points |
| --- | --- |
| Study Characteristics | Author(s) |
|  | Year |
|  | Country of publication |
|  | Sponsorship source |
|  | Primary objective |
|  | Setting |
|  | Number of Sites |
| Study Design | RCT design (e.g., parallel-group, crossover etc.) |
|  | Primary outcome |
|  | Follow-up period to primary outcome |
| Population | Inclusion/exclusion criteria |
|  | Reported methods for assessing frailty |
| Participant Characteristics | Sample Size |
|  | Age |
|  | Sex |
|  | Frailty assessment score(s) |
| Intervention and Comparator Treatment Characteristics | Type |
|  | Content |
|  | Frequency |
|  | Intensity |
|  | Volume |
|  | Duration |
|  | Setting |
| Outcomes | Number of participants retained to primary outcome (total, by group) |
|  | Number of participants retained to intervention completion |
|  | Reported reasons for attrition (number of participants impacted) |
|  | Reported strategies for maximizing retention |

**Supplementary Table S5.** Stage 2: List of excluded full-text articles and reasons for exclusion.

| **Author, Year** | **Title** | **Exclusion Reason** |
| --- | --- | --- |
| Aas 2020 | Musculoskeletal adaptations to strength training in frail elderly: a matter of quantity or quality? | Multicomponent intervention |
| Abdolhosseini 2019 | The Effects of Acute Bouts of Whole Body Vibration on Central Hemodynamics in Frail Older Adults: A Pilot Study. | Wrong study design |
| Echeverria 2019 | Effectiveness of Multicomponent Physical Exercise in old adults after hospitalization: short vs long supervised programs | Frailty not an inclusion criterion |
| Alhambra-Borrás 2019 | Effectiveness and Estimation of Cost-Effectiveness of a Group-Based Multicomponent Physical Exercise Programme on Risk of Falling and Frailty in Community-Dwelling Older Adults | Wrong study design |
| Ando 2019 | Custom-Made Daily Routine Increases the Number of Steps Taken by Frail Older Adults | Frailty not an inclusion criterion |
| Arrieta 2019 | Effects of Multicomponent Exercise on Frailty in Long Term Nursing Homes: A Randomized Controlled Trial | Frailty not an inclusion criterion |
| Bambrick 2019 | Participants' experience of a novel group exercise program: Results from the DEFRAIL (Diet and Exercise for Frailty) study pilot | Conference abstract |
| Bean 2004 | Increased Velocity Exercise Specific to Task (InVEST) training: a pilot study exploring effects on leg power, balance, and mobility in community dwelling older women | Frailty not an inclusion criterion |
| Bean 2009 | Increased velocity exercise specific to task training versus the National Institute on Aging's strength training program: changes in limb power and mobility | Frailty not an inclusion criterion |
| Benzo 2019 | Effect of home pulmonary rehab plus health coaching on copd self-management: A randomized study | Conference abstract |
| Binder 2005 | Effects of progressive resistance training on body composition in frail older adults: results of a randomized, controlled trial | Secondary study |
| Boshuizen 2005 | The effects of physical therapists’ guidance on improvement in a strength-training program for the frail elderly | Frailty not an inclusion criterion |
| Braun 2019 | An augmented prescribed exercise program (APEP) to improve mobility of older acute medical patients - a randomized, controlled pilot and feasibility trial | Frailty not an inclusion criterion |
| Cadore 2014 | Positive effects of resistance training in frail elderly patients with dementia after long-term physical restraint | Wrong study design |
| Cameron 2013 | A multifactorial interdisciplinary intervention reduces frailty in older people: randomized trial | Multicomponent intervention |
| Cesari 2015 | A physical activity intervention to treat the frailty syndrome in older persons, results from the LIFE-P study | Frailty not an inclusion criterion |
| Chan 2012 | A pilot randomized controlled trial to improve geriatric frailty | Multicomponent intervention |
| Chan 2015 | Training Intervention in a Controlled Population of Frail Elderly | Duplicate |
| Chan 2017 | Integrated care for geriatric frailty and sarcopenia: a randomized control trial | Multicomponent intervention |
| Chandler 1998 | Is lower extremity strength gain associated with improvement in physical performance and disability in frail, community-dwelling elders? | Didn’t assess frailty with a standardized measure |
| Chu 2019 | Feasibility and outcomes in a pilot randomized controlled trial of a home-based integrated physical exercise and bladder training program versus usual care for community-dwelling older women with urinary incontinence | Frailty not an inclusion criterion |
| Daniel 2012 | WiiHab for prefrail older adults | Frailty not an inclusion criterion |
| DaSilva 2019 | Obstructive sleep apnea severity and resisted exercise - Effect in aged patients - A randomized clinical trial | Conference abstract |
| DeLasHeras 2019 | Tele-rehabilitation program in idiopathic pulmonary fibrosis | Conference abstract |
| Drey 2012 | Effects of strength training versus power training on physical performance in prefrail community-dwelling older adults | Frailty not an inclusion criterion |
| Ehrari 2018 | Effects of Playful Exercise of Older Adults on Balance and Physical Activity: a Randomized Controlled Trial | Frailty not an inclusion criterion |
| Ehsani 2003 | Attenuation of cardiovascular adaptations to exercise in frail octogenarians | Secondary study |
| Faber 2006 | Effects of exercise programs on falls and mobility in frail and pre-frail older adults: a multicenter randomized controlled trial | Majority pre-frail |
| Fairhall 2012 | Effect of a multifactorial interdisciplinary intervention on mobility-related disability in frail older people: randomised controlled trial | Multicomponent intervention |
| Fairhall 2014 | Effect of a multifactorial, interdisciplinary intervention on risk factors for falls and fall rate in frail older people: a randomised controlled trial | Secondary study |
| Fu 2015 | Effectiveness of exergaming training in reducing risk and incidence of falls in frail older adults with a history of falls | Frailty not an inclusion criterion |
| Furtado 2020 | The mediating effect of different exercise programs on the immune profile of frail older women with cognitive impairment | Could not access |
| Giné-Garriga 2013 | The effect of functional circuit training on self-reported fear of falling and health status in a group of physically frail older individuals: a randomized controlled trial | Secondary study |
| Gomes 2018 | Feasibility, safety, acceptability, and functional outcomes of playing Nintendo Wii Fit PlusTM for frail older adults: A randomized feasibility clinical trial | Majority pre-frail |
| Gray 2019 | Imaging Biomarkers of the Effects of a Mixed Exercise Program | Protocol paper |
| Gudlaugsson 2012 | Effects of a 6-month multimodal training intervention on retention of functional fitness in older adults: a randomized-controlled cross-over design | Frailty not an inclusion criterion |
| Hagedorn 2010 | Effects of traditional physical training and visual computer feedback training in frail elderly patients. A randomized intervention study. | Frailty not an inclusion criterion |
| Hauer 2003 | Two years later: a prospective long-term follow-up of a training intervention in geriatric patients with a history of severe falls | Frailty not an inclusion criterion |
| Helbostad 2004 | Home training with and without additional group training in physically frail old people living at home: effect on health-related quality of life and ambulation | Didn’t assess frailty with a standardized measure |
| Herrero 2018 | Individualized Exercise Program Plus Behavioral Change Enhancement Strategies for Managing Fatigue in Frail Older People | Protocol paper |
| Hess 2006 | Ankle force and rate of force production increase following high intensity strength training in frail older adults | Frailty not an inclusion criterion |
| Hoogeboom 2010 | Preoperative therapeutic exercise in frail elderly scheduled for total hip replacement: a randomized pilot trial | Prehabilitation |
| Hsieh 2019 | Individualized home-based exercise and nutrition interventions improve frailty in older adults: a randomized controlled trial. | Majority prefrail |
| Ikezoe 2005 | Low intensity training for frail elderly women: Long-term effects on motor function and mobility | Frailty not an inclusion criterion |
| Izumi 2019 | The effect of balance exercise assist robot | Protocol paper |
| Izumi 2018 | The verification of training effect with a balance exercise assist robot on motor functions and frail | Protocol paper |
| Jeon 2014 | Effects of a randomized controlled recurrent fall prevention program on risk factors for falls in frail elderly living at home in rural communities | Frailty not an inclusion criterion |
| Karssemeijer 2019 | Exergaming as a Physical Exercise Strategy Reduces Frailty in People With Dementia: A Randomized Controlled Trial. | Frailty not an inclusion criterion |
| Kasim 2020 | Tai Chi is an effective form of exercise to reduce markers of frailty in older age. | Frailty not an inclusion criterion |
| Kaushal 2019 | Investigating dose response effects of multimodal exercise programs on health-related quality of life in older adults | Secondary study |
| Kim 2012 | Effects of exercise and amino acid supplementation on body composition and physical function in community-dwelling elderly Japanese sarcopenic women: a randomized controlled trial | Frailty not an inclusion criterion |
| Kim 2015 | Effects of exercise and milk fat globule membrane (MFGM) supplementation on body composition, physical function, and hematological parameters in community-dwelling frail Japanese women: a randomized double blind, placebo-controlled, follow-up trial | Multicomponent intervention |
| Kito 2019 | Positive Effects of Textured Lunches, Gatherings and Oral Exercises Combined with Physical Exercises on Oral and Physical Function in Older Individuals: a Cluster Randomized Controlled Trial | Frailty not an inclusion criterion |
| Koch Rysoe 2019 | Resistance Exercise Training at Different Intensities | Protocol paper |
| Kryger 2007 | Resistance training in the oldest old: consequences for muscle strength, fiber types, fiber size, and MHC isoforms | Frailty not an inclusion criterion |
| Kwon 2015 | Effects of a combined physical training and nutrition intervention on physical performance and health-related quality of life in prefrail older women living in the community: a randomized controlled trial | Frailty not an inclusion criterion |
| Lee 2013 | Effects of a multifactorial fall prevention program on fall incidence and physical function in community-dwelling older adults with risk of falls | Frailty not an inclusion criterion |
| Lee 2018 | The effects of fall prevention static and dynamic trunk stabilization exercises on the balance of elderly females | Could not access |
| Liao 2019 | Using Kinect based training to improve cognitive function and brain activation in frail and prefrail older adults: A randomized controlled trial | Duplicate |
| Liberman 2020 | Effect of Multimodal Prehabilitation vs Postoperative Rehabilitation on 30-Day Postoperative Complications for Frail Patients Undergoing Resection of Colorectal Cancer: A Randomized Clinical Trial | Duplicate |
| Liu 2018 | A Twice-Daily Individual Targeted Exercise Program in Frail Hospitalised Older Medical In-patients (RCT) | Frailty not an inclusion criterion |
| Luger 2016 | Effects of a home-based and volunteer-administered physical training, nutritional, and social support program on malnutrition and frailty in older persons: a randomized controlled trial | Multicomponent intervention |
| Lustosa 2011 | Impact of resistance exercise program on functional capacity and muscular strength of knee extensor in pre-frail community-dwelling older women: a randomized crossover trial | Frailty not an inclusion criterion |
| Luukinen 2006 | Prevention of disability by exercise among the elderly: a population-based, randomized, controlled trial | Frailty not an inclusion criterion |
| Maki 2019 | The effect of pulmonary training for community-dwelling frail elderly with chronic stroke: a randomized controlled pilot trial | Protocol paper |
| Makizako 2017 | Effects of a community disability prevention program for frail older adults at 48‚Äêmonth follow up | Wrong study design |
| Martínez Arnau  2019 | Physical Fitness, Body Composition and Frailty in Elderly People Exercise Program Effects EXERNET Elder 3.0 | Protocol paper |
| McCullagh 2020 | Augmented exercise in hospital improves physical performance and reduces negative post hospitalization events: a randomized controlled trial | Frailty not an inclusion criterion |
| McIssac 2020 | The PREPARE Trial: exercise Before Surgery to Improve Recovery in Older People With Frailty | Protocol paper |
| McMurdo 1995 | A randomized controlled trial of a home exercise programme for elderly people with poor mobility | Frailty not an inclusion criterion |
| Migliarese 2017 | Fighting frailty in underserved communities | Wrong study design |
| Ng 2015 | Nutritional, physical, cognitive, and combination interventions and frailty reversal among older adults: a randomized controlled trial | Majority prefrail |
| Oh 2017 | Effects of an integrated health education and elastic band resistance training program on physical function and muscle strength in community-dwelling elderly women: Healthy Aging and Happy Aging II study | Frailty not an inclusion criterion |
| Oosting 2012 | Preoperative home-based physical therapy versus usual care to improve functional health of frail older adults scheduled for elective total hip arthroplasty: a pilot randomized controlled trial | Prehabilitation |
| Ozaki 2017 | Training with a balance exercise assist robot is more effective than conventional training for frail older adults | Majority prefrail |
| Ozic 2020 | Interventions aimed at loneliness and fall prevention reduce frailty in elderly urban population. | Frailty not an inclusion criterion |
| Peterson 2007 | Effect of telephone exercise counseling on frailty in older veterans: project LIFE | Frailty not an inclusion criterion |
| Pollock 2012 | Whole-body vibration in addition to strength and balance exercise for falls-related functional mobility of frail older adults: a single-blind randomized controlled trial | Frailty not an inclusion criterion |
| Purdie 2019 | Tai chi to prevent falls in older adults | Could not access |
| Cuesta-Vargas  2018 | Strength training in fragile and pre-frail elderly patients at Hospital SaeoVicente de Paulo, Passo Fundo, RS: clinical trial | Protocol paper |
| Rejeski 2008 | Physical activity in prefrail older adults: confidence and satisfaction related to physical function | Frailty not an inclusion criterion |
| Rezola-Pardo 2019 | Comparison between multicomponent and simultaneous dual-task exercise interventions in long-term nursing home residents: the Ageing-ONDUAL-TASK randomized controlled study | Frailty not an inclusion criterion |
| Rosendahl 2006 | High-intensity functional exercise program and protein enriched energy supplement for older persons dependent in activities of daily living: a randomised controlled trial | Frailty not an inclusion criterion |
| Rosie 2007 | Sit-to-stand as home exercise for mobility-limited adults over 80 years of age: GrandStand System TM may keep you standing? | Frailty not an inclusion criterion |
| Saeterbakken 2018 | Effects of strength training on muscle properties, physical function, and physical activity among frail older people: a pilot study | Frailty not an inclusion criterion |
| Sato 2007 | The water exercise improves health-related quality of life of frail elderly people at day service facility | Didn’t assess frailty with a standardized measure |
| Seino 2017 | Effects of a multifactorial intervention comprising resistance exercise, nutritional and psychosocial programs on frailty and functional health in community-dwelling older adults: A randomized, controlled, cross-over trial | Multicomponent intervention |
| Serra-Prat 2017 | Effectiveness of an intervention to prevent frailty in pre-frail community-dwelling older people consulting in primary care: a randomised controlled trial | Frailty not an inclusion criterion |
| Serra Rexach 2011 | Short-term, light-to moderate-intensity exercise training improves leg muscle strength in the oldest old: a randomized controlled trial | Frailty not an inclusion criterion |
| Suikkanen 2019 | Older persons with signs of frailty in a home-based physical exercise intervention: baseline characteristics of an RCT. | No outcome data |
| Takano 2017 | Differences in the effect of exercise interventions between prefrail older adults and older adults without frailty: A pilot study | Frailty not an inclusion criterion |
| Tarazona- Santabalbina 2015 | Lifestyle Intervention Trial in Obese Elderly | Multicomponent intervention |
| Tarazona-Santabalbina 2015 | Physical Training During Hospital Admission With Community-Acquired Pneumonia | Protocol paper |
| Tatsuya 2019 | Effects of Inspiratory Muscle Training in Frail elderly people on Cough Strength | Could not access |
| TCTR20190528002 2019 | The effects of cognitive and exercise training on cognitive and physical functions in older adults with different types of frailty - Exploration of the elderly with physical frailty alone and cognitive frailty | Could not access |
| Timmons 2015 | Intervention Assessing the Role of Exercise Program and Nutrition Supplement for Sarcopenia | Frailty not an inclusion criterion |
| Timonen 2002 | Effects of a group-based exercise program on the mood state of frail older women after discharge from hospital | Frailty not an inclusion criterion |
| Tousignant 2013 | Efficacy of supervised Tai Chi exercises versus conventional physical therapy exercises in fall prevention for frail older adults: a randomized controlled trial | Didn’t assess frailty with a standardized measure |
| Vestergaard 2008 | Home-based video exercise intervention for community-dwelling frail older women: a randomized controlled trial | Frailty not an inclusion criterion |
| Villareal 2004 | Effects of exercise training on bone mineral density in frail older women and men: a randomised controlled trial | Secondary study |
| Villareal 2005 | Weight Loss and Exercise in Obese, Physically Limited, Older Women and Men | Wrong study design |
| Villareal 2005 | Weight Loss and Exercise in Physically Limited Obese Elderly Subjects | Duplicate |
| Villareal 2006 | Effect of weight loss and exercise on frailty in obese older adults | Multicomponent intervention |
| Vicente-Rodríguez  2019 | Impact of a Multicomponent Exercise Program on Functional Capacity in Frail Aged Participants With Cognitive Decline | Protocol paper |
| Waite 2017 | Home-based preoperative rehabilitation (prehab) to improve physical function and reduce hospital length of stay for frail patients undergoing coronary artery bypass graft and valve surgery | Prehabilitation |
| Watt 2011 | Effect of a supervised hip flexor stretching program on gait in frail elderly patients | Didn’t assess frailty with a standardized measure |
| Westhoff 2000 | Effects of a low-intensity strength-training program on knee-extensor strength and functional ability of frail older people | Frailty not an inclusion criterion |
| Wherry 2019 | Feasibility of a Home-Based Balance Intervention in Middle-Aged Women Using Wii Fit Plus | Frailty not an inclusion criterion |
| Wolf 1996 | Reducing frailty and falls in older persons: an investigation of Tai Chi and computerized balance training | Frailty not an inclusion criterion |
| Wolf 2003 | Intense tai chi exercise training and fall occurrences in older, transitionally frail adults: a randomized, controlled trial | Frailty not an inclusion criterion |
| Wolf 2006 | The influence of intense Tai Chi training on physical performance and hemodynamic outcomes in transitionally frail, older adults | Frailty not an inclusion criterion |
| Wollesen 2018 | Physical functioning, cognition and psychosocial well-being for elderly, multimorbid nursing home residents | Protocol paper |
| Worm 2001 | Effects of a multicomponent exercise program on functional ability in community-dwelling, frail older adults | Didn’t assess frailty with a standardized measure |
| Yamada 2012 | Community-based exercise program is cost-effective by preventing care and disability in Japanese frail older adults | Wrong study design |
| Yamada 2017 | Self-management group exercise extends healthy life expectancy in frail community-dwelling older adults | Wrong study design |
| Yuki 2019 | Standardization and verification of exercise for sarcopenia -randomized controlled trial- | Could not access |
| Zech 2012 | Residual effects of muscle strength and muscle power training and detraining on physical function in community-dwelling prefrail older adults: a randomized controlled trial | Frailty not an inclusion criterion |

**Supplementary Table S6.** Summary of included studies.

| Author (Year)  Country  RCT Design  Number of Centres | Inclusion Criteria | Intervention  (Frequency, Intensity, Type, Volume, Duration, Setting) | Control  (Frequency, Intensity, Type, Volume, Duration, Setting) |
| --- | --- | --- | --- |
| Binder (2002)  USA  Parallel group  n=1 | (1) score between 18 and 32 on the modified PPT, (2) report of difficulty or need for assistance with up to two IADLs or one ADL, or (3) achievement of a O2 peak between 10 and 18 mL kg1 min1 | F: 3x/wk  I: 65-100% 1RM  T: Multicomponent – Phase 1: 22 exercises (flexibility, balance, strength); Phase 2: progressive resistance training (knee extension/ flexion, bench press, row, leg press, biceps curl, abdominals); Phase 3: endurance (treadmills, stationary bikes, aerodyne bikes, rowing machines)  V: Phase 1: NR; Phase 2: 1-3 sets, 6-12 reps; Phase 3: 15-30 min  D: 12 weeks  S: Exercise facility at a medical school | F: 2-3x/wk  I: Low intensity  T: Multicomponent - 9 of the 22 exercises included in phase 1 of the intervention program completed at home  (mainly flexibility). Also attended a 1hr training session at exercise facility and group exercise class 1x/month.  V: 1 hour training session at beginning followed by at-home sessions  D: 9 months  S: Home/Exercise facility at a medical school |
| Brown (2000)  USA  Parallel group  n=1 | Sedentary men and women over the age of 78 years who were living independently, who scored score less than 32points on the PPT. | F: 3x/wk  I: Low intensity  T: Multicomponent – 22 exercises (flexibility, balance, strength)  V: NR  D: 3 months  S: Outpatient rehabilitation | F: NR  I: NR  T: Flexibility – 9 of the 22 exercises performed in the intervention group). Also attended an onsite exercise session 1x/month.  V: NR  D: 3 months  S: Home |
| Cadore (2014)  Spain  Parallel group  n=1 | Adults who were 85 years or older and met Fried's criteria for frailty. | F: 2x/wk  I: 40-60% 1RM  T: Multicomponent – Resistance (leg/knee extension, seated bench press); Balance/gait (semi-tandem foot standing, line walking, stepping, walking with obstacles, proprioceptive exercises, altering base of support and weight transfer)  V: Resistance: 8-10 reps; Overall: 40 min (5 min warmup, 10 min balance/gait, 20 min resistance, 5 min stretching)  D: 12 weeks  S: NR | F: 4x/wk  I: NR  T: Flexibility – small active and passive movements as a series of stretched in a rhythmic fashion to individual joints (routinely encouraged in most Spanish nursing homes)  V: 30 min  D: 12 weeks  S: NR |
| Chan (2017)  China  Parallel group, wait-list control  n=4 | Age 65 or above, scored 5 or above in the Tilburg Frailty Index, physically fit to sit on a chair and cognitively competent to understand instructions from the practitioner and to sign the consent form (scored 6 or above in Abbreviated Mental Test Score) | F: 4x/wk  I: NA  T: Acupressure – acupressure protocol to the face, head and shoulders (delivered 2x/wk by registered Chinese medicine practitioner, 2x/wk by caregiver)  V: 15 min  D: 12 weeks  S: Neighbourhood elderly centers | F: NR  I: NR  T: Usual care  V: NR  D: 12 weeks  S: NR |
| Clegg (2014)  England  Parallel group, pilot RCT  n=1^†^ | People living at home and under the care of a case manager or community matron; the housebound; attending a day centre or respite care; residence in assisted living sites; at discharge from intermediate care hospitals and following attendance at elderly medicine outpatient clinics in Bradford, UK. | F: 3x/day, 5x/week  I: NR  T: Multicomponent – Strength (Breathing,  sitting-up, spine rotation, armchair rise‚ arm strength, leg kicks, toe-heel pointing, marching,  standing arm raises,  armchair rise, calf raises, leg swing back, side stepping, wall press-up, leg back swing and side raise, stand on one leg, walking toe to heel); Aerobic exercises (varied based on level of function)  V: Progress from 5-15 reps, <15min total  D: 12 weeks  S: Home | F: NR  I: NR  T: Usual care – Treatment from primary care team  V: NR  D: 12 weeks  S: Home |
| Furtado (2020)  Portugal  Intervention Arm 1  Parallel group, 3 arms  n=1^†^ | Participants were institutionalized-dwelling women living in centres of health and social support (CHS) in the city of Coimbra, Portugal Participants had to be 70 years old or more, be frail or pre-frail, clinically stable with their drug therapy updated. | F: 2-3x/wk  I: Low to moderate (50-75% HRM, BORG RPE 1-5)  T: Multicomponent – Warm up (body mobilization/ dynamic flexibility exercises); Workout (walking/mobility: walking, chair-based sit and reach, easy skipping, arms coordinator, chair-based leg extension and overhead reach,  coordinator, balance, quickness, agility integrated exercise, standing rear leg extension); Cool down (body mobilization and static flexibility exercises)  V: 45min  D: 28 weeks  S: Centres of health and social support | F: NA  I: NA  T: Non-exercise – The control group did not participate in any physical exercise intervention but was encouraged to maintain their normal routine, which included a monthly agenda of artistic and cultural activities  V: NA  D: NA  S: Centres of health and social support |
| Furtado (2020)  Portugal  Intervention Arm 2  Parallel group, 3 arms  n=1 | Participants were institutionalized-dwelling women living in centres of health and social support (CHS) in the city of Coimbra, Portugal Participants had to be 70 years old or more, be frail or pre-frail, clinically stable with their drug therapy updated. | F: 2-3x/wk  I: Low to moderate (55-80% HRM, OMNI RPE 1-6)  T: Multicomponent – Warm up (body mobilization/ dynamic flexibility exercises); Workout (chair-based strength training: front squat, unilateral hip flexion, bench over row (with flexion), chest press, standing reverse fly, chair spine twist extension arm, shoulder press twist arm front position, frontal total raised, biceps arm curl, overhead triceps extension; Cool down (body mobilization and static flexibility exercises)  V: 45min  D: 28 weeks  S: Centres of health and social support | F: NA  I: NA  T: Non-exercise – The control group did not participate in any physical exercise intervention but was encouraged to maintain their normal routine, which included a monthly agenda of artistic and cultural activities  V: NA  D: NA  S: Centres of health and social support |
| Gill (2002)  USA  Parallel Group  n=1^†^ | Physically frail, elderly persons 75 years of age or older from busy primary care practices in southern Connecticut | F: Balance (daily) conditioning (3x/wk)  I: NR  T: Multicomponent - Progressive exercise (ROM, balance, and muscle conditioning/ strengthening using thera-band elastic bands); Education (instruction in safer, more effective mobility techniques providing training in the proper use of assistive devices and making recommendations for environmental modifications; recommendations were also provided for problems with feet/footwear or impairments in sensation/tone)  V: 40min (balance (10), conditioning (30)  D: 6 months  S: Home | F: 1x/month  I: NA  T: Attention and health education - a health educator and the participant reviewed general practices promoting good health, such as proper nutrition, management of medications, physical activity, sleep hygiene, and other health-related areas; 19 Sessions were tailored to the participant’s specific needs  V: NR  D: 6 months  S: Home |
| Gine- Garriga (2010)  Spain  Parallel Group  n=1 | Individuals 80-90 years of age with physical frailty. | F: 2x/week (balance (1x/wk), strength (1x/wk))  I: Strength (BORG RPE 12-14)  T: Multicomponent - Balance (Static (one and two-leg balance with toes or heels raised and tandem standing w/ eyes open or closed), dynamic (walking on different surfaces, elevations, performing dual task and incorporating different gait pattens and speed) and functional balance exercises (walking with obstacles, semi-dark environment, obstructed view of the feet, picking up objects)); Strength (rising from a chair, stair climbing, knee bends, floor transfer, lunges, leg squat, leg extension, leg flexion, calf  raise, and abdominal curl using ankle weights)  V: Balance: 4 sets; Strength 1-2 sets, 6-15 reps  D: 12 weeks  S: Primary care facility | F: 1x/wk  I: NA  T: Multicomponent – Routine care; Socialization (with researchers and other participants); Education sessions (within socialization sessions)  V: 60min  D: 12 weeks  S: Primary care facility/  home |
| Kubicki (2014)  France  Parallel Group  n=1 | Frail, balance disorder and can remain standing without any mechanical or human help. | F: 2x/wk  I: NR  T: Exergaming -  2-D virtual reality-based game (a yellow ball appeared on a screen and after a short variable delay and in a random position. Patients were asked to react as soon as possible and to reach their arm to the ball as fast as possible)  V: 10 sequences  D: 3 weeks  S: Research lab | F: 3x/wk  I: NR  T: Classical Rehabilitation  V: NR  D: 3 weeks  S: NR |
| Langlois (2013)  Canada  Parallel group, wait-list control  NR | Frail and non-frail older adults. | F: 3x/wk  I: Moderate (BORG RPE 0-10)  T: Multicomponent – Warm up (stretching and balancing); Aerobic workout (treadmill, recumbent bike, elliptical); Strength training, cool-down.  V: 1 hour (warm-up: 10 min; aerobic: 10-30min; strength training: 10-min; cool down: 10-min)  D: 12 weeks  S: NR | F: NA  I: NA  T: Usual care - Instructed to maintain current level of activity during the study period - offered intervention after the control period  V: NA  D: 12 weeks  S: NR |
| Latham (2003)  USA  Parallel group, 4 arms (2x2 factorial design)  n=5 | 65 and older, considered frail according to simple clinical measures of frailty as described by Winograd et al., 19 and no clear indication or contraindication to either of the study treatments (i.e., the clinician had substantial uncertainty about the benefits of the treatments for a specific patient). | F: 3x/week  I: 60-80% 1RM  T: Strength Training - Quadriceps exercise program (warm up stretches (e.g., hamstring, quad stretches), exercise: knee extensions with adjustable ankle cuff weights)  V: 3 sets, 8 reps  D: 10 weeks  S: Hospital and home | F: 3x/week  I: NA  T: Attention control - frequency matched telephone calls and home visits from the research physical therapist. During these contacts, the research physical therapist inquired about the patients’ recovery, gave general advice on any problems encountered, and supported appropriate actions taken toward recovery.  V: NA  D: 10 weeks  S: Home |
| Liao (2019)  Taiwan  Parallel group  n=5 | (1) age between 65 to 90 years old and (2) the presence of at least one of the 5 Fried Criteria. | F: 3x/wk  I: 50-75% HRM, RPE 12-14 (somewhat hard)  T: Exergaming - Part 1: Warm up (Tai Chi), balance, stability, and muscle exercises (moving arms/legs in  circular motions, weight shifting, squatting). Part 2: Resistance and aerobic exercises (PAPAMAMA program used for upper/lower extremities). Part 3: Balance game (Participants needed to: reach toward a target in a given location quickly and accurately; lift their legs to cross obstacles or to climb up and down steps at a predetermined speed)  V: 60 min  D: 12 weeks  S: NR | F: 3x/wk  I: 50-75% HRM, RPE 12-14 (somewhat hard)  T: Multicomponent - Resistance training (shoulders (abductors, adductors and rotators), elbows (flexors and  extensors), and wrists and fingers (flexors and extensors). Lower extremities - emphasized muscles  that are important for balance and gait control)); Aerobic (stepping exercises  in the seated and standing positions, stepping on/off of a stool, standing up from and sitting in a chair); Balance (dynamic and static).  V: 60 min  D: 12 weeks  S: NR |
| Mollindeo Cardalda  (2019)  Spain  Intervention Arm 1  Cluster RCT  n=3 | (a) Frail-aged adults, (b) aged over 75 years, (c) diagnosed with mild to moderate cognitive impairment, (d) able to stand and walk for at least 30m without shortness of breath (e) able to walk safely and independently without aid (f) resident of a geriatric long-term care home | F: 2x/wk  I: NR  T: Strength training - Lower limb strength training (warm up (mobility of ankle, knee, hip); strength exercises (plantar and dorsal flexors, flexors and knee extensors, flexors, extensors, abductors and hip rotators); cool-down)  V: 60min (2-3 sets, 10-15 reps)  D: 12 weeks  S: Residential care home | F: NA  I: NA  T: Usual care (Crafts, reading comprehension  and cognitive stimulation)  V: NA  D: NA  S: 12 weeks |
| Mollindeo Cardalda  (2019)  Spain  Intervention Arm 2  Cluster RCT  n=3 | (a) Frail-aged adults, (b) aged over 75 years, (c) diagnosed with mild to moderate cognitive impairment, (d) able to stand and walk for at least 30m without shortness of breath (e) able to walk safely and independently without aid (f) resident of a geriatric long-term care home | F: 2x/wk  I: NR  T: Range of Motion - Traditional physical exercise program designed for institutionalized elderly people, aimed at increasing range of mobility and coordination, specifically focused on the lower limbs,  performed mostly in the seated position. Exercises that were performed in the standing position required assistance.  V: 60min  D: 12 weeks  S: Residential care home | F: NA  I: NA  T: Usual care - Crafts, reading comprehension  and cognitive stimulation  V: NA  D: 12 weeks  S: Residential care home |
| Mugueta- Aguinaga (2017)  Spain  Parallel group (pilot RCT)  n=2 | Persons over 65 years of age with a Barthel score equal to or above 90 points who carry out no scheduled physical activity. | F: 3x/week  I: NR  T: Exergaming – interactive video game (First part targeted upper and lower extremities, while the second/third targeted upper and lower  extremities. The game entails several scenarios, with each one representing one or more steps in a simplified process. The user starts performing the different activities in order and operated by remote control, with each activity referring to a specific movement of the upper and/or lower extremity)  V: 20 min  D: 3 weeks  S: Residential home | F: NR  I: NR  T: Routine care - continued with “daily life”  V: NR  D: 3 weeks  S: Residential home |
| Mugueta- Aguinaga (2019)  Spain  Parallel group  n=2 | Persons over 65 years of age with a Barthel score equal to or above 90 points who carry out no scheduled physical activity. | F: 3x/week  I: NR  T: Exergaming – interactive video game (First part targeted upper and lower extremities, while the second/third targeted upper and lower  extremities. The game entails several scenarios, with each one representing one or more steps in a simplified process. The user starts performing the different activities in order and operated by remote control, with each activity referring to a specific movement of the upper and/or lower extremity)  V: 20min  D: 6 weeks  S: Elderly day center | F: NA  I: NA  T: Usual care - continued with their daily lives with no physical activity scheduled  V: NA  D: 6 weeks  S: NA |
| Kessler (2014)  Switzerland  Parallel group  n=2 | Aged over 65 years, able to stand with or without aids, a score of at least 16 points in the mini mental test (MMT) and a score of <6 points in the SPPB. | F: 3x/week  I: Passive  T: Stochastic Resonance Whole Body Vibration – Participants stood on a platform with slightly bent hip, knee and ankle joints without shoes and were allowed to hold on to bars at each side. They vibrated with a frequency of 3 Hz and noise level of 4. They progressed vibration frequency to 6 Hz and the parallel standing position to tandem standing up and slow functional squats.  V: 10 min (5 x 1 min vibration period with 1 min break between sets)  D: 4 weeks  S: Retirement home | F: 3x/week  I: Passive  T: Sham Treatment - The control group vibrated with a basic frequency of 1 Hz and noise level 1 with no increase of the basic frequency and no additional exercises.  V: 10 min (5 min vibration period with 1 min break between sets)  D: 4 weeks  S: Retirement home |
| Paw (2001)  The Netherlands  Parallel group, 4 arms (2x2 factorial design)  n=1 | Age 70 years or older, frail, care services required (eg, home care, Meals-on-Wheels), non-institutionalized, no terminal disease or rapidly deteriorating health status, no multivitamins taken in the past month, and an ability to comprehend the study procedures. | F: 2x/week  I: Moderate (6-8 on 10-point RPE)  T: Multicomponent - Warm up (walking, exercise to music routines, familiarization with equipment); Skills training (apply physiologic parameters (strength, speed, flexibility, coordination, endurance) to perform and sustain motor actions (reach, throw, catch, kick, chair stand, bend down, toe/heel raise) and learn to apply behaviours in games and cooperative activities); Cool down (stretching and relaxing activities)  V: 45 min  D: 17 weeks  S: NR | F: Bi-weekly  I: NA  T: Attention Control - Social program (lectures, social activities, crafts). On alternating weeks, they were visited at their home and given a supply of fresh food products.  V: 90 min  D: 17 weeks  S: NR |
| Rydwik (2008)  Sweden  Parallel group, 4 arms  NR | a) unintentional weight loss ‚â• 5% and/or body mass index (BMI) ‚â§20 kg/m2; and b) low physical activity level (grade 3 on a six-graded scale of physical activity during the last six months, present cancer treatment, stroke within the last two years, less than 7 points of a total 9-point score on the short form of the Mini Mental State Examination, and institutionalization. | F: 2x/week  I: 60-80% 1RM  T: Multicomponent - Warm-up (aerobic training: standing exercises - walking/jogging on the spot, walking forwards/backwards/ sideways and arm movements); Muscle strength-training (leg press, dips, pull-down on stationary equipment and functional strength training (chair stand, step-up and toe raise)); Balance (Qigong exercises on different supporting area combined with arm and trunk movements)  V: 1 hour (Warm-up: 20 min; Muscle strength-training: 20 min; Balance: 20 min)  D: 12 weeks  S: NR | F: NR  I: NA  T: Education - General exercise advice (walk 3x/week for >20min, to use staircases vs elevator and to follow WHO recommendation of 30min of PA/day); Diet advice (3 main courses and 2-3 snacks including meat, fish, egg, fruit and vegetables, dairy products and fibre in combination with fluid each day)  V: NR  D: 12 weeks  S: Home |
| Tarazona-Santabalbina  (2016)  Spain  Parallel group  n=2 | Men and women aged 70 years or older who were (1) sedentary (less than 3 hours of weekly physical activity), (2) frail according to the frailty phenotype, (3) with a gait speed slower than 0.8 m/s, (4) and were community dwellers. | F: 5x/week  I: Aerobic (40-65% HRM), Strength (25-75% 1RM)  T: Multicomponent - Proprioception and balance exercises (postural sway and dynamic balance, coordination, and flexibility of the lumbo-pelvic area), aerobic training (walking around a circuit and climbing stairs), strength training (resistance bands and included isometric, concentric, and eccentric exercises with arms, hands, and legs), stretching (arms, legs and neck)  V: 65 min  D: 24 weeks  S: Primary rural care center | F: NA  I: NA  T: Usual care - No physical training, attended regular primary care program  V: NA  D: 24 weeks  S: Primary rural care center |
| Villareal (2011)  USA  Parallel group, 4 arms  n=1 | 65 years of age or older and obese (BMI of 30 or more), if they had a sedentary lifestyle, if their body weight had been stable during the previous year (i.e., had not fluctuated more than 2 kg), and if their medications had been stable for 6 months before enrollment. All participants had to have mild-to-moderate frailty. | F: 3x/week  I: Aerobic (65-85% HR Peak), Strength (65-80% 1RM)  T: Multicomponent - Aerobic exercises (walking on a treadmill, stationary cycling, and stair climbing); Resistance training (9 upper and lower-extremity exercises with the use of weight-lifting machines); Flexibility and balance exercises  V: 90 min (1-3 sets, 8-12 reps of resistance training exercises)  D: 52 weeks  S: NR | F: 1x/month  I: NA  T: Usual Care + Education - Did not receive advice to change their diet or activity habits and were prohibited from participating in any weight-loss or exercise program. They were provided general information about a healthy diet during monthly visits with the staff.  V: NR  D: 52 weeks  S: NR |
| Wadsworth (2020)  New Zealand  Intervention Arm  Parallel group  NR | Frailty, as determined by functional ambulation using a scale of 0-6, where a score of 0-5 indicates a degree of functional limitation | F: 3x/week  I: NR  T: Whole Body Vibration + Standard Residential Care – Participants stood with isometric knee flexion during bouts of vibration, interspersed with rest periods. Progression of Hz and/or amplitude, up to a maximum of 26 Hz/4.0 mm, were self-determined by participants.  V: 10-20 min (5-10 x 1 min vibration period with 1 min break between sets)  D: 16 weeks  S: Residential care facilities | F: NR  I: NR  T: Standard care  V: NR  D: 16 weeks  S: Residential care facilities |
| Wadsworth (2020)  New Zealand  Sham Treatment Arm  Parallel group  NR | Frailty, as determined by functional ambulation using a scale of 0-6, where a score of 0-5 indicates a degree of functional limitation | F: 3x/week  I: NR  T: Simulated Whole-Body Vibration + Standard Care – Participants mimicked the stance and duration of the intervention arm.  V: 10-20 min (5-10 x 1 min vibration period with 1 min break between sets)  D: 16 weeks  S: Residential care facilities | F: NR  I: NR  T: Standard care  V: NR  D: 16 weeks  S: Residential care facilities |

Abbreviations –NR – Not Reported

^†^ Represents a city or region

**Supplementary Table S7.** Summary of study characteristics and retention rates.

| Author (Year) | Intervention  n  Age, mean (SD) or median (1^st^, 3^rd^)  Female (%)  Frailty Measurement Tool (Score) | Control  n  Age, mean (SD) or median (1^st^, 3^rd^)  Female (%)  Frailty Measurement Tool (Score) | Intervention  Enrolled (N),  Retained to primary outcome (n_1_)  Retained to intervention completion (n_2_) | Control  Enrolled (N),  Retained to primary outcome (n_1_)  Retained to intervention completion (n_2_) |
| --- | --- | --- | --- | --- |
| Binder (2002) | 66  83(4)  34 (52)  OARS instrument, Physical Function subscale of FSQ (25.9 (4.0)), Modified PPT (28.4 (4.7)), Peak O^2^ Uptake (15.4 (2.9)) | 49  83(4)  26 (53)  OARS instrument, Physical Function subscale of FSQ (28.3 (5.9)), Modified PPT (28.3 (4.7)), Peak O^2^ Uptake (15.6 (2.6)) | 69  66 (96%)  46 (67%) | 50  49 (98%)  41 (82%) |
| Brown (2000) | 48  83 (4)  28 (62)  PPT (29(4)) | 39  83 (4)  22 (56)  PPT (29(6)) | NR | NR |
| Cadore (2014)* | 16  93 (3)  8 (50)  Fried Frailty Phenotype (NR) | 16  90 (1)  10 (63)  Fried Frailty Phenotype (NR) | 16  11 (69%)*  11 (69%) | 16  13 (81%)*  13 (81%) |
| Chan (2017) | 54  76 (7)  36 (67)  TFI (7.13 (1.76) | 52  76 (8)  38 (73)  TFI (7.22 (1.78)) | 54  NR  NR  Overall:  106  NR  79 (75%) | 52  NR  NR |
| Clegg (2014) | 45  79 (8)  33 (73)  TUG Test (50.9 (62.0), EFS (7.8 (2.4)) | 39  78 (11)  27 (69)  TUG Test (51.2 (66.2), EFS (8.3 (2.7)) | 45  40 (89%)  28 (62%) | 39  30 (77%)  30 (77%) |
| Furtado (2020)*  Intervention Arm 1 | 28  80 (8)  28 (100)  Fried Frailty Phenotype  2.35 (1.42) | 28  81 (10)  28 (100)  Fried Frailty Phenotype  2.37 (1.19) | 28  21 (75%)  24 (86%) | 28  19 (68%)  19 (68%) |
| Furtado (2020)*  Intervention Arm 2 | 28  81 (5)  28 (100)  Fried Frailty Phenotype  2.38 (1.72) | 28  81 (10)  28 (100)  Fried Frailty Phenotype  2.37 (1.19) | 28  20 (71%)  22 (79%) | 28  19 (68%)  19 (68%) |
| Gill (2002) | 94  83 (5)  80 (85)  Rapid gait test (NR), chair stand (NR) | 94  84 (5)  70 (75)  Rapid gait test (NR), chair stand (NR) | 94  88 (94%)  61 (65%) | 94  90 (96%)  78 (83%) |
| Gine- Garriga* (2010) | 26  84 (3)  13 (50)  Fried Frailty Phenotype (NR) | 25  84 (3)  12 (48)  Fried Frailty Phenotype  (NR) | 26  22 (85%)  22 (85%) | 25  19 (76%)  19 (76%) |
| Kubicki (2014) | 23  82 (7)  18 (78)  Fried Frailty Phenotype (NR) | 23  82 (5)  17 (74)  Fried Frailty Phenotype (NR) | 23  18 (78%)  18 (78%) | 23  14 (61%)  14 (61%) |
| Langlois (2013) | 17  75 (7)  12 (71)  Fried Frailty Phenotype (NR) | 17  75 (5)  13 (77)  Fried Frailty Phenotype (NR) | 17  14 (82%)  14 (82%) | 17  15 (88%)  15 (88%) |
| Latham (2003) | 120  80 (79-81) ^‡^  66 (55)  Winograd et al. criteria (NR) | 123  78 (77-80) ^‡^  63 (51)  Winograd et al. criteria (NR) | 120  112 (93%)  NR | 123  110 (89%)  NR |
| Liao*  (2019) | 31  80 (9)  19 (61)  Fried Frailty Phenotype (3.0 (1.3)) | 30  84 (6)  17 (57)  Fried Frailty Phenotype (3.0 (1.4)) | 31  27 (87%)  27 (87%) | 30  25 (83%)  25 (83%) |
| Mollindeo Cardalda*  (2019)  Intervention Arm 1 | 30  86 (8)  20 (67)  Fried Frailty Phenotype (NR) | 30  85 (7)  19 (63)  Fried Frailty Phenotype (NR) | 13.43  11.20 (83%)  11.20 (83%) | 13.43  12.99 (97%)  12.99 (97%) |
| Mollindeo Cardalda  (2019)  Intervention Arm 2 | 30  84 (8)  16 (53)  Fried Frailty Phenotype (NR) | 30  85 (7)  19 (63)  Fried Frailty Phenotype (NR) | 13.43  10.30 (77%)  10.30 (77%) | 13.43  12.99 (97%)  12.99 (97%) |
| Mugueta- Aguinaga* (2017) | 20  86 (7)  12 (60)  SPPB (7.25 (1.86)) | 20  83 (9)  11 (55)  SPPB (7.16 (1.07)) | 20  20 (100%)  20 (100%) | 20  19 (95%)  20 (100%) |
| Mugueta- Aguinaga (2019) | 20  86 (7)  12 (60)  SPPB (7.28 (1.86)) | 20  83 (9)  12 (60)  SPPB (7.16 (1.07)) | 20  20 (100%)  20 (100%) | 20  19 (95%)  20 (100%) |
| Kessler* (2014) | 14  91 (8)  8 (57)  SPPB (3 (2-4.5) | 13  84 (9)  8 (62)  SPPB (4 (2-6) | 14  13 (93%)  13 (93%) | 13  11 (85%)  13 (100%) |
| Paw* (2001) | 39  76 (5)  28 (72)  Fried Frailty Phenotype (NR) | 37  79 (7)  25 (68)  Fried Frailty Phenotype (NR) | 55  NR  NR  Overall:  217  161 (74%)  161 (74%) | 55  NR  NR |
| Rydwik (2008) | 23  84 (4)  11 (48)  Fried Frailty Phenotype (NR) | 23  83 (4)  16 (70)  Fried Frailty Phenotype (NR) | 23  20 (87%)*  23 (100%) | 23  19 (83%)  23 (100%) |
| Tarazona-Santabalbina  (2016) | 51  80 (4)  29 (57)  Fried Frailty Phenotype (3.6 (0.8)) | 49  80 (4)  25 (51)  Fried Frailty Phenotype (3.8 (0.6)) | 51  40 (78%)  NR | 49  40 (82%)  NR |
| Villareal (2011) | 26  70 (4)  16 (62)  PPT (27.1 (3.1)), O^2^ Peak (17.4 (3.5)), FSQ (29.8 (3.3)) | 27  69 (4)  18 (67)  PPT (26.8 (4.5)), O^2^ Peak (16.3 (3.8)), FSQ (30.5 (3.2)) | 26  22 (85%)  22 (85%) | 27  23 (85%)  23 (85%) |
| Wadsworth (2020)  Intervention Arm | 36  79 (1)  21 (58)  FAS (5.0 (0.1) | 46  84 (1)  28 (61)  FAS (4.7 (0.2) | 36  27 (75%)  33 (92%) | 46  28 (61%)  38 (83%) |
| Wadsworth (2020)  Sham Treatment Arm | 35  84 (1)  27 (77)  FAS (5.1 (0.1) | 46  84 (1)  28 (61)  FAS (4.7 (0.2) | 35  30 (86%)  32 (91%) | 46  28 (61%)  38 (83%) |

Abbreviations – OARS – Older American Resources and Services; FSQ – Functional Status Questionnaire; PPT – Physical Performance Test; O^2^ – Oxygen; TFI – Tillburg Frailty Index; TUG – Timed Up and Go; EFS – Edmonton Frail Scale; NR – Not Reported

*Age, % female representative of patients analyzed not patients enrolled.

^‡^ Data presented as age (95% confidence interval)

**Supplementary Table S8.** Design effect calculations for Mollinedo Cardalda (2019).

|  | **Primary Outcome** | | | |
| --- | --- | --- | --- | --- |
|  | Enrolled | | Retained | |
|  | Unadjusted | Adjusted | Unadjusted | Adjusted |
| IG 1 | 30 | 13.4 | 25 | 11.2 |
| IG 2 | 30 | 13.4 | 23 | 10.3 |
| CG | 30 | 13.4 | 29 | 13.0 |
|  | **Intervention Completion** | | | |
|  | Enrolled | | Retained | |
|  | Unadjusted | Adjusted | Unadjusted | Adjusted |
| IG 1 | 30 | 13.4 | 25 | 11.2 |
| IG 2 | 30 | 13.4 | 23 | 10.3 |
| CG | 30 | 13.4 | 29 | 13.0 |

Legend: Mollinedo Cardalda et al. adopted a cluster RCT design. We calculated effective sample sizes by reducing binary outcomes by the Design Effect (DE). To calculate DE, we used the formula DE = 1 + (M – 1)(ICC) where M is the average cluster size (25.667) and ICC is the intracluster correlation coefficient. We did not identify a reported intracluster coefficient, thus we used a value of 0.05.^24^ The calculation was DE = 1 + (25.667 – 1)(0.05) = 2.233.

Abbreviations: IG: Intervention group; CG: Comparator group

**Supplementary Table S9.** Sensitivity analyses for the primary outcome (retention to primary outcome measurement).

| Study | Pooled Retention Rate | Adjusted Pooled Retention Rate |
| --- | --- | --- |
| Binder 2002 | 0.85 (0.80, 0.90) | 0.84 (0.79, 0.89) |
| Cadore 2014 |  | 0.86 (0.81, 0.90) |
| Clegg 2014 |  | 0.85 (0.80, 0.90) |
| Furtado 2020 |  | 0.86 (0.81, 0.90) |
| Gill 2002 |  | 0.85 (0.80, 0.90) |
| Gine-Garriga 2010 |  | 0.86 (0.80, 0.90) |
| Kubicki 2014 |  | 0.86 (0.81, 0.90) |
| Langlois 2013 |  | 0.85 (0.80, 0.90) |
| Latham 2003 |  | 0.85 (0.80, 0.89) |
| Liao 2019 |  | 0.85 (0.80, 0.90) |
| Mollinedo Cardalda 2019 |  | 0.85 (0.80, 0.90) |
| Mugueta-Aguinaga 2017 |  | 0.84 (0.79, 0.89) |
| Mugueta-Aguinaga 2019 |  | 0.84 (0.79. 0.89) |
| Kessler 2014 |  | 0.85 (0.80, 0.90) |
| Paw 2001 |  | 0.86 (0.81, 0.90) |
| Rydwik 2008 |  | 0.85 (0.80, 0.90) |
| Tarazona-Santabalbina 2016 |  | 0.86 (0.80, 0.90) |
| Villareal 2011 |  | 0.85 (0.80, 0.90) |
| Wadsworth 2020 |  | 0.86 (0.81, 0.90) |

Legend: Studies are included in the first column. The second column indicates the pooled retention rate for all studies that reported retention to primary outcome measurement. The third column represent sensitivity analyses. We removed one study at a time from the pooled retention rate to determine whether the results were robust to the influence of individual studies. The adjusted pooled retention rate is representative of the pooled retention rate with the removal of the study indicated in each row. No single study had a statistically significant impact on the overall pooled retention rate (all 95% confidence intervals overlap).
